# Development of evidence-based indicators for the detection of drug-related problems among ovarian cancer patients

**DOI:** 10.3389/fphar.2023.1203648

**Published:** 2023-06-30

**Authors:** Kala Bahadur Rawal, Uday Venkat Mateti, Vijith Shetty, Chakrakodi Shashidhara Shastry, Mazhuvancherry Kesavan Unnikrishnan, Shraddha Shetty, Aparna Rajesh

**Affiliations:** ^1^ Department of Pharmacy Practice, NGSM Institute of Pharmaceutical Sciences, Nitte (Deemed to be University), Mangaluru, Karnataka, India; ^2^ Department of Medical Oncology, KS Hegde Medical Academy (KSHEMA), Justice KS Hegde Charitable Hospital, Mangaluru, Karnataka, India; ^3^ Department of Biostatistics, KS Hegde Medical Academy (KSHEMA), Nitte (Deemed to be University), Mangaluru, Karnataka, India; ^4^ Department of Obstetrics and Gynecology, KS Hegde Medical Academy (KSHEMA), Justice KS Hegde Charitable Hospital, Nitte (Deemed to be University), Mangaluru, Karnataka, India

**Keywords:** Antineoplastic agents, drug safety, ovarian neoplasm, delphi, medication-related problems

## Abstract

**Background:** Antineoplastic drugs produce serious drug-related problems and their management is challenging. DRPs are critical, for saving on therapeutic costs, particularly in resource poor settings within low-middle-income countries such as India. Indicators are clues that helps to detect DRPs within the healthcare organization and minimize overall harm from medications. Indicators enable healthcare professionals to determine the future therapeutic course. And enable healthcare professionals to take a proactive stand, and stay informed and empowered to both prevent and manage DRPs. This study aims to develop evidence-based indicators for detecting potential drug-related problems in ovarian cancer patients.

**Patients and Methods:** A retrospective study was conducted in the Department of Oncology of a tertiary care teaching hospital in South India. Based on literature search, we developed a list of indicators, which were validated by a Delphi panel of multidisciplinary healthcare professionals (16 members). Based on 2 years of ovarian cancer data, we performed a feasibility test retrospectively and classified the DRPs according to the Pharmaceutical Care Network Europe classification of DRPs version-9.1.

**Results:** The feasibility test identified 130 out of 200 indicators. A total of 803 pDRPs were identified under four main categories: drug selection problem, drug use problem, adverse drug reaction and drug-drug interaction The most frequently observed were ADR 381 (47.45%), DDIs 354 (44.08%), and drug selection problems 62 (7.72%).

**Conclusion:** Indicators developed by us effectively identified pDRPs in ovarian cancer patients, which can potentially help healthcare professionals in the early detection, timely management, and attenuating severity of DRPs. Identifying the pDDIs can potentially improve interdisciplinary involvement and task sharing, including enhanced pharmacists’ participation within the healthcare team.

## 1 Introduction

Ovarian cancer is either epithelial cancer, *i.e.,* originating from the surface of the ovary, or germ cell malignant neoplasm, *i.e.,* originating from the egg cells (What is ovarian cancer?, 2022). According to the Globocan 2020 data, annual incidence and mortality from ovarian cancer worldwide were 3,13,959 cases and 2,07,252 deaths, respectively. South-Eastern Asia witnessed 31,169 new cases and 20,012 deaths yearly (Globocan, 2020: Ovary cancer, 2022). The incidence of ovarian cancer in India, one of the world’s most populated countries, was 45,701 in 2020. Ovarian cancer is the eighth most common cancer in females and the second most common gynaecological cancer in Asia (Globocan, 2020: Ovary cancer, 2022). Among Indian women, ovarian cancer is the third most common after breast and cervical cancer, and the second most common gynaecological cancer (Globocan, 2020: Ovary cancer, 2023). Apart from high incidence and prevalence, ovarian cancer was associated with 32,077 deaths in 2020, accounting for 3.8% mortality among Indian cancer patients (Globocan, 2020: Ovary cancer, 2023).

According to the cancer registry of India, the lifetime risk of Indian women getting ovarian cancer ranges from 0.9 to 8.84 per one lakh women. Although ovarian cancer has a poor prognosis and a high mortality rate, early diagnosis and judicious medical intervention show a better prognosis than in late and advanced stages (Consensus document for management of epithelial ovarian cancer, 2022). Ovarian cancer has a lower incidence but three times higher mortality than breast cancer and is the fifth most common cause of death in females (Momenimovahed et al., 2019; Key statistics for ovarian cancer, 2022). Approaches to managing ovarian cancer include surgery, radiation therapy, chemotherapy, hormonal, and targeted/immunological therapy (Treating ovarian cancer, 2023). However, the 5-year survival rate among ovarian cancer patients (revealed by SEER data) was reported to be 49.7% from 2014 to 2018 (National institute of cancer surveillance, 2022).

Despite significant advances in diagnosing and treating ovarian cancer, studying drug-related problems (DRPs) among ovarian cancer patients remains suboptimal in day-to-day clinical rotation. The risk of DRPs increases with polypharmacy, consequent to comorbidities and supportive therapy. More than 50% of elderly ovarian cancer patients experience polypharmacy because they receive a minimum of five medications (Oldak et al., 2019). DRPs are events or circumstances associated with drug therapy that potentially affect healthcare outcomes (Jayakumar et al., 2021). Complexity in cancer treatment extends beyond anticancer agents because comorbidities demand supportive therapy. A specialized pharmacist called an oncology pharmacist can initiate prescription audits, recommend deprescribing, minimize polypharmacy and reduce the potential harms due to therapy (Yokoyama et al., 2018).

The incidence of DRPs in ovarian cancer is unknown in India. Antineoplastic drugs produce serious DRPs, and their management is challenging because of their narrow therapeutic index (Sisay et al., 2015; Degu et al., 2017). In contrast to the therapeutic benefits of medicine, DRPs can increase morbidity and mortality (van Roozendaal and Krass, 2009). Unresolved and under-resolved DRPs can result in needless hospitalization, readmission, extended hospital stay, and extended care. DRPs will not only impact therapeutic efficacy, but also raise treatment costs.^15^ *.* Various studies have highlighted that patients’ safety is a crucial and continuous process (Oldak et al., 2019; Jayakumar et al., 2021), and one of the vital factors impacting patients’ safety is DRPs (Yokoyama et al., 2018; Jayakumar et al., 2021). A study focusing on DRPs in ovarian cancer is needed, which can give information regarding the incidence of DRPs in ovarian cancer and make the healthcare provider aware of the possible DRPs risk.

Reports suggest that 25% of hospital admissions of cancer patients possibly result from DRPs (Sisay et al., 2015), of which 50% were potentially preventable with timely intervention (Degu et al., 2017). DRPs may compromise patients’ physical health and health-related quality of life to a large extent, leading to a significant waste of healthcare expenditures (Ni et al., 2021). DRPs are critical, for saving on therapeutic costs, particularly in limited resource settings within low-middle income countries such as India (Dror et al., 2008; Sisay et al., 2015).

Indicators are clues that help detect DRPs within the healthcare organization and minimize overall harm from medications (van Roozendaal and Krass, 2009; Thiyagu et al., 2010). This approach is based on identifying and addressing the errors that are associated with adverse therapeutic outcomes. Indicators offer an approach to standardizing error identification that may provide more consistent and accurate information. Indicators enable healthcare professionals and patients to determine the future therapeutic course. Indicators enhance healthcare professionals to take a proactive stand, and stay informed and empowered to both prevent and manage DRPs (MacKinnon et al., 2008; van Roozendaal and Krass, 2009). With the help of developed indicators, the healthcare provider can identify DRPs and recommend to the prescriber what action plan could be implemented in the next steps to prevent or resolve potential DRPs. and timely identification of DRPs could prevent patients from possible harm. It is critical to optimize management by identifying and preventing DRPs (MacKinnon et al., 2008; Thiyagu et al., 2010).

The present study aimed to design and develop novel, evidence-based indicators for detecting DRPs among ovarian cancer patients to improve drug safety and promote positive clinical outcomes.

## 2 Patients and methods

### 2.1 Study design and ethical approval

This retrospective study was conducted in the Department of Oncology of a Tertiary Care Teaching Hospital in South India. The study was initiated after obtaining approval from the central ethics committee (Ref. no. NU/CEC/2021/143) and was registered in the clinical trial registry of India (Ref. No: CTRI/2021/08/035818).

### 2.2 Development of indicators

The list of evidence-based indicators was developed through a literature review that included primary sources (original research articles, case studies, and case series), secondary sources (databases like; UpToDate, Micromedex, review articles, and systematic reviews), and tertiary sources (Textbooks, and Guidelines (European Society for Medical Oncology (ESMO), American Society of Clinical Oncology (ASCO), National Comprehensive Cancer Network (NCCN)) [Supplementary Materials S1, S2].

### 2.3 Validation of the indicators

Multidisciplinary health professionals validated the indicators in three stages. The Delphi panel consisted of 16 validators, consisting of three oncologists, two oncopharmacists, two oncology nurses, two gynecologists, one general medicine physician, one general surgery physician, two clinical pharmacists, and three academic pharmacists (van Roozendaal and Krass, 2009; Thiyagu et al., 2010; Lecours, 2020).

In the first stage, a preliminary list of indicators was prepared by the authors. An independent oncopharmacist outside the Delphi panel subsequently scrutinized this list. After scrutiny, all the Delphi members were asked individually (directly approached) for their voluntary participation in validation process, and after the Delphi members accepted the request, the above list was sent to the Delphi panel, who independently scored each indicator (on a Likert scale from 1 to 5) based on the perceived relevance of each quality attribute. Furthermore, the members of the Delphi panel were also asked to supply additional indicators along with their comments. The Delphi panel members were also asked to give their personal opinion on indicators that scored less than three on the Likert scale (van Roozendaal and Krass, 2009; Thiyagu et al., 2010).

In the second stage, the list of additionally suggested indicators listed by individual members of the Delphi panel was sent back to the Delphi panel, enabling members to re-evaluate scores given by other members. Similarly, the Delphi panel was also asked to comment and re-evaluate the scores allotted to each indicator based on the scores given by other members (van Roozendaal and Krass, 2009; Thiyagu et al., 2010).

In the third validation stage, we computed the average of all scores given to each indicator. Those indicators scoring less than three points were eliminated from the indicators list (van Roozendaal and Krass, 2009; Thiyagu et al., 2010).

### 2.4 Confidentiality of the delphi panel

The confidentiality of the individual members of Delphi panel was maintained throughout the study in order to guarantee respect for expert opinion, facilitate privacy and prevent scoring bias. Written informed consent was obtained from all members of the Delphi panel, with the right to withdraw consent at any stage during the study (Lecours, 2020; The Delphi method techniques and applications, 2022).

### 2.5 Feasibility testing of the indicators

**FIGURE 1:**
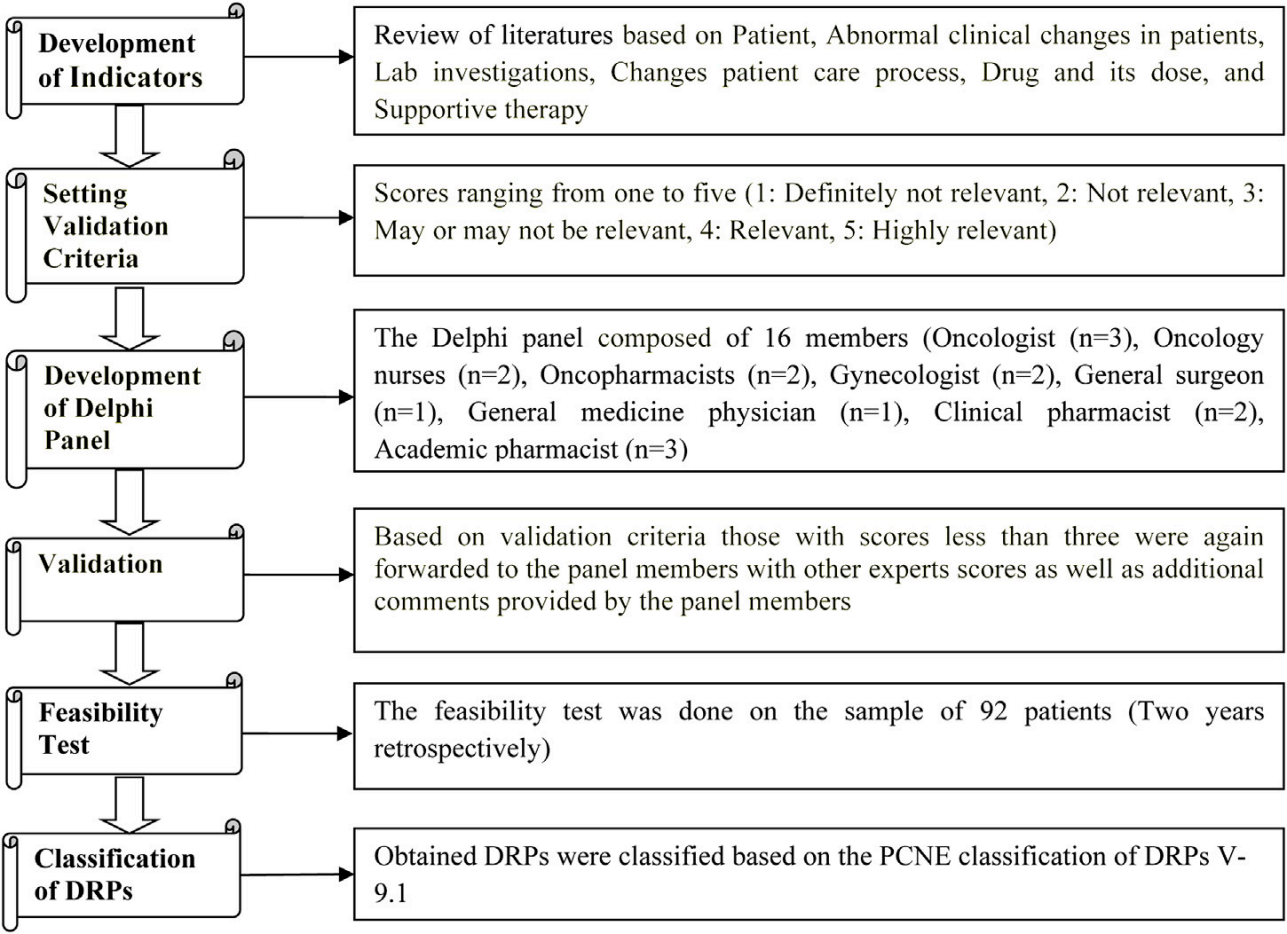
Study procedure.

The feasibility test of indicators was performed using 2 years data retrospectively from 2019 to 2020 in 92 patients from the Medical Records Department. The obtained DRPs were classified based on the Pharmaceutical Care Network Europe (PCNE) classification of DRPs V 9.1 (van Roozendaal and Krass, 2009; Thiyagu et al., 2010; Classification for Drug related problems V9.1, 2022) [Figure 1].

### 2.6 Inclusion and exclusion criteria

The study included ovarian cancer patients (aged 18 years and above) who underwent chemotherapy, targeted therapy, and hormonal therapy. Those who underwent radiation therapy, surgery and those with incomplete files were excluded.

### 2.7 Sample size

This study sample size was calculated by taking the standard deviation (*σ* = 1.22) of DRPs occurrence from cases of gynecological cancer (cervical cancer) (Degu et al., 2017). At 95% confidence interval, and 0.25 margin of error (d) the required sample size was calculated to be 92 ovarian cancer patients.

### 2.8 Data collection

The data collection form was designed to include study-relevant information such as socio-demographic details (age, weight, height, BMI, domiciliary status), comorbidities, past medical and medication history, personal history, social habits, drug utilization patterns and patient complaints after administration of the therapy. The pharmacist screened and identified DRPs from patients’ case sheets which were confirmed by the treating physician.

### 2.9 Statistical analysis

The data collected were analyzed using the SPSS version 20. The quantitative data were expressed in terms of descriptive statistics (age, height, BSA, weight, BMI, gravida status, last childbirth, number of drugs prescribed), whereas qualitative data (qualification, occupation, personal history, family history, social history, social classes, domiciliary status, marital status, menstrual history) were expressed as frequency.

## 3 Results

### 3.1 Indicators

A total of 190 indicators were developed, covering three aspects of therapy viz, antineoplastic therapy, supportive therapy, and patient drug adherence. The indicators were sent for validation by the Delphi Panel. Out of 190 indicators developed, 178 got a score three or above. The 12 indicators with less than score three were sent back to the Delphi panel for second step of validation. In addition to validating the 12 indicators with suboptimal scores, the Delphi panel suggested 13 additional indicators, amounting to a total of 25 indicators.

Of the 25 indicators, 15 got scores three or above, and 10 got below three. The 10 indicators with suboptimal scores were sent back to the Delphi panel for the third validation in which 7 got scores three and above. Finally, the 200 indicators with scores three or above were selected for the study [Supplementary Material S2].

### 3.2 Patient demographics

Of the, 92 patients (aged 18–75 years) included in this study, mean age was 50.1 ± 11.7 years 86 (93.5%); had epithelial ovarian cancer, of whom 52 (56.5%) were in stage III C, 58 (63%) were under adjuvant therapy, and 51 (55.4%) received first-line of cancer therapy. Most of the patients were in the second 26 (28.3%) and third 23 (25%) cycle of chemotherapy. 30 (32.6%), patients had comorbidities, of which hypertension was the most common 16 (17.4%) [Table 1].

**TABLE 1:** Baseline characters.

Age		
Age in range (Years)	Number of patients	Percentage (%)
18–24	3	3.3
25–34	8	8.7
35–44	17	18.5
45–54	28	30.4
55–64	27	29.4
65–74	8	8.7
75 and above	1	1.1
**Mean Weight (kg)**	51.7 ± 10	
**Body Mass Index (BMI)**		

**BMI in Range**	**Number of patients**	**Percentage**
Below 18.5	10	10.9
18.5 to 24.9	63	68.5
25 to 29.9	13	14.1
30 to 34.9	4	4.4
35 to 39.9	1	1.1
Above 40	1	1.1
Mean ± SD	22.6 ± 4.5	
**Marital status**		

**Marital status**	**Number of patients**	**Percentage**
Married	88	95.7
Unmarried	4	4.4
**Domiciliary status**		
Rural	52	56.5
Urban	40	43.5
**Comorbidities**		
Hypertension	16	17.4
Hypothyroidism	4	4.4
Diabetes mellitus	3	3.3
Hypertension and hypothyroidism	1	1.1
Hypertension and hyperthyroidism	1	1.1
Rheumatic arthritis	2	2.2
Ischemic heart disease	2	2.2
Gastric ulcer	1	1.1
Total comorbidities	30	32.6

### 3.3 Drug use among the patients

A total of 1,173 medications were in use among the patients. Antiemetics and gastroprotectants were the most frequently used drugs. 318 antiemetics drugs were prescribed, followed by gastroprotectants (*n* = 221) and antineoplastic agents (*n* = 212). From antiemetics ondansetron and dexamethasone were administered in all patients. Similarly, ranitidine was most frequently used gastroprotectant.

At the time of hospital admission, all patients were prescribed five or more drugs (6–14 drugs), with a mean of 9.8 drugs per patient. During discharge, more than 50% of the patients were prescribed more than five medications with a mean of 5.3 drugs per patient [Table 2].

**TABLE 2:** Frequency of drug use among patients.

**Category of drug**	**Frequency (n=1173)**	**Percentage**
**Antineoplastic agents**	**212**	18.1
**A. Chemotherapeutic agents**	189	16.1
**Natural products**	80	6.8
Paclitaxel	57	4.9
Doxorubicin	10	0.9
Etoposide	7	0.6
Bleomycin	5	0.4
Vincristine	1	0.1
**Alkylating agents**	86	7.3
Carboplatin	77	6.6
Cisplatin	7	0.6
Cyclophosphamide	1	0.1
Ifosfamide	1	0.1
**Antimetabolites**	23	2.0
Gemcitabine	23	2.0
**B. Targeted drug therapy**	23	2.0
Bevacizumab	23	2.0
**C. Antiemetics**	318	27.1
**NK1 receptor antagonist**	13	1.1
Aprepitant	12	1.0
Fosaprepitant	1	0.1
**D2 antagonist**	11	0.9
Domperidone	11	0.9
**5HT3 receptor antagonist**	168	14.3
Ondansetron	92	7.8
Palonosetron	76	6.5
**Glucocorticoids**	92	7.8
Dexamethasone	92	7.8
**Prokinetic agents**	3	0.3
Metoclopramide	3	0.3
**Benzo diazepam**	31	2.6
Lorazepam	31	2.6
**D. Gastro protectant**	221	18.8
**Proton pump inhibitors**	20	1.7
Pantoprazole	20	1.7
**H2-receptor antagonists**	90	7.7
Ranitidine	90	7.7
**Others**	111	9.5
Domperidone+ Rabeprazole	87	7.4
Antacids	9	0.8
Laxative	14	1.2
Ulcer protectant	1	0.1
**E. Anti-allergic**	96	8.2
Levocetirizine	4	0.3
Pheniramine	84	7.2
Hydrocortisone	8	0.7
**E. Analgesic**	40	3.4
Paracetamol	3	0.3
Tramadol	2	0.2
Naproxen	1	0.1
Diclofenac	1	0.1
Paracetamol and Tramadol	33	2.8
**F. Analgesic and Anxiolytics**	4	0.3
Gabapentin and Nortriptyline	3	0.3
Pregabalin+ Nortriptyline	1	0.1
**G. Antitussive**	6	0.5
Phenylephrine+ Chlorpheniramine Maleate+ Dextromethorphan Hydrobromide	5	0.4
Levocetirizine+ Montelukast	1	0.1
**H. Antibiotic**	27	2.3
**I. Granulocyte colony stimulating factor**	77	6.6
Filgrastim	69	5.9
Pegfilgrastim	8	0.7
**J. Supplements**	126	10.7
**Electrolytes**	18	1.5
Calcium and Vitamin D3	4	0.3
Sodium	1	0.1
Potassium	7	0.6
Magnesium	5	0.4
**Nutritional Supplements**	108	9.2
Folic acid	14	1.2
Iron and Folic acid	9	0.8
Iron Folic acid and Vitamin B12	9	0.8
Vitamin Complex	41	3.5
Vitamin Complex and Vitamin C	2	0.2
Pregabalin and B12	8	0.7
Amino acid+ Vitamin B12+ Pregabalin	3	0.3
Multivitamin+ Minerals	5	0.4
Protein	17	1.4
**K. Antihypertensive**	22	1.9
Β-blocker	2	0.2
Calcium channel blocker	6	0.5
Angiotensin converting enzyme inhibitors	3	0.3
Angiotensin receptor blockers	3	0.3
Diuretics	8	0.7
**L. Others**	24	2.0
Methylprednisolone	2	0.2
Disodium Hydrogen citrate	2	0.2
Levosalbutamol	4	0.3
Oxygen Supply,	2	0.2
Red blood cell transfusion	2	0.2
Metronidazole	1	0.1
Loperamide	1	0.1
Hydroxychloroquine,	1	0.1
Hyoscine butyl bromide	1	0.1
Levothyroxine	4	0.3
Carbimazole	1	0.1
Amitriptyline	1	0.1
Mirtazapine	2	0.2
**Drugs prescribed in admission time**	**Number of drugs**	
Minimum	6	
Maximum	14	
Mean± SD	9.8±1.8	
**Drugs prescribed during discharge time**	**Number of drugs**	
Minimum	3	
Maximum	9	
Mean± SD	5.3±1.2	

*5HT3: 5-hydroxytryptamine 3 receptor antagonist, NK1 receptor; Neurokinin 1 receptor

### 3.4 Drug-related problems

The total potential DRPs were found to be 803, out of which the most common pDRPs were ADRs 381 (47.5%), followed by the potential drug-drug interactions (pDDIs) 354 (44.1%). In which this study observed proportion of 8.73 pDRPs per patient, 4.1 ADRs per patients, and 3.54 pDDIs per patients [Table 3].

**TABLE 3:** Potential drug-related problems in study sample.

DRPs	Number of DRPs (n = 803)	Percentage	Proportion per patients (n = 92)
**1. Drug selection problem**	62	7.7	0.67
1.1. Drug duplication	35	4.4	0.38
1.2. Therapy without indications	25	3.1	0.27
1.3. Many drugs for one indication	2	0.3	0.02
**2. Drug use problem**	6	0.8	0.07
**3. ADRs**	381	47.5	4.14
**4. pDDIs**	354	44.1	3.85
Total	803	100	8.73

*DRPs, Drug-related problems; ADRs, Adverse drug reactions; pDDIs, Potential drug-drug interactions.

#### 3.4.1 Drug selection problem

This category of DRPs were recorded 62. The most commonly observed were drug duplication 35 (4.4%) followed by therapy without indication 25 (3.1%) and many drugs for one indication 2 (0.3%). Of the drug selection problems, the inappropriate duplication of active ingredients of vitamin B12 was found in 23 (25%) patients. Seven patients (7.6%) received multiple drugs of the same class viz pantoprazole and rabeprazole. Similarly, 5 patients (5.4%) were prescribed antacids containing the same active ingredients of aluminum and magnesium hydroxide. Under therapy without indications, 8 patients (8.7%) were prescribed an analgesic, E.g., paracetamol and tramadol without complaints of pain, and 10 patients (10.9%) were prescribed anti-allergic medication without complaints of sore throat, urticaria or runny nose. In case of too many medications for one indication, 2 patients (2.2%) were prescribed more than three antihypertensives for the same indication [Table 3].

#### 3.4.2 Drug use problem

Six patients (6.5%) were prescribed with the second dose of the drug aprepitant 80mg, but it was not administered [Table 3].

#### 3.4.3 Adverse drug reactions

Out of 381 ADRs, the most frequently detected ADRs were alopecia (*n* = 75; 19.7%), nausea and vomiting (*n* = 63; 16.5%), and high blood pressure (*n* = 37; 9%). Alopecia, nausea, and vomiting were commonly observed with platinum, taxel, and anthracycline agents. Hypertension was most commonly reported with vascular endothelial growth factor inhibitors (VEGFi). [Table 3].

#### 3.4.4 Potential drug-drug interactions

Polypharmacy, to the extent of an average of 9.3 drugs, was observed in patients. An increase in the number of prescribed drugs also increased the risk of drug-drug interactions. All observed interactions were pDDIs, which were not clinically evident but could potentially cause adverse events. Out of the 354 pDDIs, the most frequently observed were between domperidone and ondansetron (n = 74; 20.9%), paclitaxel and carboplatin (n = 58; 16.4%), domperidone and tramadol (n = 34; 9.6%) and ondansetron and tramadol (n = 27; 7.4%) [Table 3].

## 4 Discussion

Indicators provide signals for the identification and management of DRPs by supporting quick detection, risk assessment, causative link determination, decision assistance, monitoring and evaluation, quality improvement, and fostering interdisciplinary communication and collaboration. By utilizing indicators, healthcare providers can improve patient safety, drug therapy, and outcomes. The Delphi approach was used in this study to validate the developed indicators. Through this Delphi approach, we may gather the most trustworthy consensus view on specific indicators from panel members in a multistage interactive session (Lecours, 2020; The Delphi method techniques and applications, 2022).

This study has developed a list of evidence-based indicators to identify DRPs in ovarian cancer patients and helped in detecting a significant number of DRPs. These DRPs were classified under four classes based on the PCNE classification of DRPs V9.1 (van Roozendaal and Krass, 2009; Classification for Drug related problems V9.1, 2022). The mean of DRPs per patient in our study was 8.7, which is significantly higher than the values reported in Kenya (2.65 ± 1.22 DRPs per cervical cancer patient) possibly because we evaluated all the treatment cycles of patients for one full year (Degu et al., 2017). Early detection and assessment of DRPs is a crucial step that may help mitigate the adverse effects of the DRPs and facilitate timely management (van Roozendaal and Krass, 2009; Yeoh et al., 2015). Collectively, patients undergoing antineoplastic therapy will have higher chances of DRPs because patients undergoing anticancer therapy are prescribed more than five drugs during admission and discharge (Sisay et al., 2015). For instance, our inpatient prescriptions had an average of 9.8 ± 1.8 drugs, and discharge prescriptions had an average of 5.3 ± 1.2 drugs.

Likewise, more than 60% and 80% of the gynecological cancer patients underwent polypharmacy in Kenya and in USA Odak S studies respectively (Degu et al., 2017; Oldak et al., 2019). As the number of drugs increases, the risk of DRPs also increases proportionately. Ovarian cancer patients are always at risk of polypharmacy, consistent with the study findings (Oldak et al., 2019).

We faced drug selection issues (the most frequently encountered issue) 62, comprising (7.7%) of the total DRPs. This is comparatively less than what was reported in cervical cancer 64 (29.8%) (Degu et al., 2017) and among cancers in general 92 (24.1%) (Sisay et al., 2015). Duplication of the active ingredients was the most common problem in drug selection 35 (38.1%), especially the duplication of vitamin B12 in multivitamins/other formulations. Absorption of vitamin B12 takes place through facilitated diffusion in the distal portion of the ileum, with the help of a transport protein called intrinsic factor. Overdose of vitamin B12 leads to saturation of intrinsic factors, simultaneously leading to the oligo absorption of vitamin B12, thus resulting in therapeutic failure and progression to megaloblastic anemia and peripheral neuropathy (Brahmkar and Jaishwal, 2015; Advantages and disadvantages of protein assisted transport, 2022).

Additionally, 7 patients were prescribed two proton pump inhibitors (pantoprazole and rabeprazole) and 5 patients were prescribed two antacid syrups containing the same active ingredients, namely, aluminium hydroxide and magnesium hydroxide. Using two drugs with the same mechanism concomitantly could lead to overdose, with increased adverse effects rather than therapeutic benefits. This, in turn, adds to the economic burden from unnecessary additional doses as well as the therapeutic burden from potential adverse effects of an overdose (Kumar and Rajasekhar, 2020).

Significant reasons for polypharmacy include the prescription of drugs without indication and the prescription of too many drugs for one indication. Both contribute to therapeutic complexity, DDIs, ADRs, and economic burden (Oldak et al., 2019; Kumar and Rajasekhar, 2020).

Under drug selection issues, the drug without indication was observed in 23 (25%) patients. On the other hand, 2 patients (2.2%) were prescribed 4 antihypertensives for a single indication, called multimodal therapy, potentially leading to severe hypotension (Taking multiple medicines safely, 2023).

In our study, 6 patients (6.3%) did not receive the second prescribed dose of aprepitant 80 mg, (24 h after the first dose of aprepitant 125 mg) to mitigate chemotherapy-induced nausea and vomiting (Ritchie and Kohli, 2022).

The most frequently observed ADRs were alopecia 75 (81.5%) and nausea and vomiting 65 (68.5%). Correspondingly, the results of an Indian study showed a similar predominance of alopecia and nausea and vomiting at 85.3% and 65%, respectively (Ingale et al., 2021). Likewise, reports from a Bangladesh study showed the incidence of alopecia (58%) and nausea and vomiting (52%) to be the most common (Poddar et al., 2010).

Most patients were prescribed highly emetogenic drugs like taxel, platinum derivatives, doxorubicin, and cyclophosphamide. Around 16 (17.4%) experienced itching or sensitivity issues with carboplatin therapy and blood transfusion. In our study, 15 (16.3%) patients experienced pain at the injection site and/or thrombophlebitis. Likewise, reports by the Korean study, advanced-stage cancer patients undergoing parenteral antineoplastic therapy were treated with topical local analgesics (Lee et al., 2019).

Following alopecia and nausea and vomiting, (12 patients (13%) had experienced diarrhea/constipation. A similar incidence of constipation was observed in 12.3% of patients in North East India study (Wahlang et al., 2017). Our results differ from the above study results in the Nepali population, wherein around 54% of patients had experienced constipation and diarrhea (Shrestha et al., 2017). The major reason for this could be the use of antineoplastics agents. Constipation could result from the concomitant use of antiemetics (5HT3 antagonist) and opioid analgesic. Diarrhea could be the result of laxatives.

The stress from chemotherapy could be the reason for the rise in blood pressure (BP) in our study (*n* = 37; 40.2%). Additionally, antineoplastic agents, steroids (dexamethasone) as antiemetic, *etc.*, could also raise blood pressure. The temporary rise of BP was managed by psychosocial counseling and making the patients comfortable, whereas chronic BP was treated by antihypertensive as well as other palliative care. Hypertension was more prevalent in targeted therapy involving angiogenesis inhibitors (Mouhayar and Salahudeen, 2011).

Another frequently observed ADR was hematological disorders 39 (42.4%), dominantly anemia 13 (14.1%) followed by leucopenia 8 (8.7%), leukocytosis (7 (7.6%), eosinophilia 6 (6.52%) and thrombocytopenia (3 (3.3%) patients. Similar incidence of hematological disorder has been reported in 40.5% of the patients in Nepali populations (Mallik et al., 2007). Whereas, neutropenia was found only in two patients in our study, possibly due to the effective use of the granulocyte-colony stimulating factor (G-CSF) (Gupta et al., 2010).

Here, all listed drug-drug interactions in our study were potential interactions rather than actual ones. However, we need to be watchful so that we do not miss ADRs (van Leeuwen et al., 2015). Along with the anticancer agents, patients are also prescribed with supportive therapy to outweigh the risk of adverse effects of anticancer agents. As the number of drugs increase in the prescription, it makes the therapy more complex and increases the risk of interactions between drugs. Drug-drug interactions may result in negative outcomes either by suppressing the therapeutic effects of one drug by another drug or by promoting the toxic effects of another drug (Kannan et al., 2011; Riechelmann and Girardi, 2016).

In this study, we have observed 3.9 pDDIs per patient, which is similar to results from South India and Pakistan with 2.8 and 2.7 pDDIs per patient respectively (Kannan et al., 2011; Ismail et al., 2020). In the present study 75 pDDIs were observed among antineoplastic agents alone or with supportive therapy. Most commonly observed pDDIs were with paclitaxel and carboplatin, possibly due to frequent prescriptions. Platinum derivatives could cause pDDIs if administered before taxel, possibly because altered serum concentration can enhance myelosuppression by taxel. In case of supportive therapy, pDDIs were observed 279 times. Ondansetron and domperidone were the most common (*n* = 74) followed by domperidone and tramadol (*n* = 34) and tramadol and ondansetron (*n* = 27). Co-administration of ondansetron and domperidone may lead to QT-prolongation, necessitating frequent electrocardiograms (ECG) and monitoring of signs and symptoms for palpitations or arrhythmias. However, ondansetron and domperidone combination have a therapeutically superior antiemetic effect. Moreover, a combination of tramadol with domperidone or ondansetron may increase the risk of serotonin syndrome (Kamath et al., 2021).

## 5 Strengths and limitations of the study

### 5.1 Strength

As far as we know, this study represents the first attempt to develop indicators for identifying DRPs among ovarian cancer patients. Our methodology involves the participation of a multidisciplinary healthcare team in which pharmacists would also play a significant supportive role. There is a dearth of investigations analysing DRPs in most cancers, particularly ovarian cancer in Indian patients, and much of this is because the physician is overworked and the non-physician members of the healthcare team play a suboptimal role. Pharmacotherapy in India does not optimally elicit the participation of pharmacists and other paramedical professionals. India has a poor allopathic doctor: patient ratio (1 doctor per 1,194), which is below the WHO recommended 1: 1,000 (Sharma et al., 2013; Ghosh, 2022). This study highlights the potential role of pharmacists in enhancing the capability of an inclusive healthcare team by offering a supporting role by minimizing DRPs and augmenting therapeutic success. In other words, our study focuses on a fresh strategy that invokes the principle of task sharing, as envisaged by WHO and thereby expanding patients’ therapeutic experience (Orkin et al., 2021). Highlight the pDRPs of ovarian cancer treatment, provide early alerts and provide precautionary measures for mitigating inappropriate drug use, in addition to saving on healthcare expenses.

### 5.2 Limitations

Being observational rather than interventional, this study has analysed the pDRPs rather than the actual DRPs. Had it been interventional, DRPs could have been identified in real time and managed appropriately and promptly. Having prepared the indicators with a focus on Indian population, the results do not automatically apply to cover the rest of the world populations.

## 6 Conclusion

Cancer treatment being highly complex, expensive, multidisciplinary, and extremely risky, therapy will benefit immensely from identifying DRPs which can potentially simplify preventive strategies by spotting real world problems at the point of care (Yokoyama et al., 2018; Oldak et al., 2019). Since most of the DRPs are preventable, an early detection can help to mitigate and treat them promptly and abort the incidence of adverse outcomes (Degu et al., 2017). Having adopted the Delphi approach towards identifying evidence-based indicators for DRPs, the study has incorporated multiple viewpoints and the cumulative experiences of a variety of interdisciplinary professionals in cancer therapy (Orkin et al., 2021). An extensive range of DRPs were identified from the indicator, namely, drug selection problems, drug use problems, ADRs, and DDIs. Data generated by our study may also be deployed as a training strategy for familiarizing healthcare professionals with the idea of task sharing and team work.

## Data Availability

The original contributions presented in the study are included in the article/Supplementary Material, further inquiries can be directed to the corresponding author.

## References

[B1] Advantages and disadvantages of protein assisted transport (2022). Advantages and disadvantages of protein assisted transport. Available from: https://uta.pressbooks.pub/cellphysiology/chapter/advantages-and-disadvantages-of-protein-assisted-transport/ (Accessed November 14th, 2022).

[B2] Brahmkar D. M. Jaishwal S. B. (2015). Absorption of drugs. Biopharmaceutics and pharmacokinetics- A treatise 3rd edition. Delhi: Vallabh Parkashan, p5–p97.

[B3] Classification for Drug related problems V9.1 (2022). Classification for Drug related problems V9.1. Available from: https://www.pcne.org/upload/files/417_PCNE_classification_V9-1_final.pdf (Accessed November 14th, 2022).

[B4] Consensus document for management of epithelial ovarian cancer (2022). Consensus document for management of epithelial ovarian cancer. Available from: https://main.icmr.nic.in/sites/default/files/guidelines/Ovarian_Cancer.pdf (Accessed November 14th, 2022).

[B5] Degu A. Njogu P. Weru I. Karimi P. (2017). Assessment of drug therapy problems among patients with cervical cancer at Kenyatta National Hospital, Kenya. Gynaecol. Oncol. Res. Pract. 4, 15. 10.1186/s40661-017-0054-9 PMC5648473 29075505

[B6] Dror D. M. van Putten-Rademaker O. Koren R. (2008). Cost of illness: Evidence from a study in five resource-poor locations in India. Indian J. Med. Res. 127 (4), 347–361. 18577789

[B7] Ghosh A. (2022). India's doctor-patient ratio better than WHO norm of 1:1000, government tells Rajya Sabha the Print 2022. Available from: https://theprint.in/health/indias-doctor-patient-ratio-better-than-who-norm-of-11000-government-tells-rajya-sabha/1273080/ (Accessed April 10th, 2023).

[B8] Globocan 2020: Ovary cancer (2022). Globocan 2020: Ovary cancer. Available from: https://gco.iarc.fr/today/data/factsheets/cancers/25-Ovary-fact-sheet.pdf (Accessed November 14th, 2022).

[B9] Globocan 2020: Ovary cancer (2023). Globocan 2020: Ovary cancer. Available from: https://gco.iarc.fr/today/data/factsheets/populations/356-india-fact-sheets.pdf (Accessed February 23rd, 2023).

[B10] Gupta S. Singh P. K. Bhatt M. L. Pant M. C. Gupta R. Negi M. P. (2010). Efficacy of granulocyte colony stimulating factor as a secondary prophylaxis along with full-dose chemotherapy following a prior cycle of febrile neutropenia. Biosci. Trends 4 (5), 273–278. 21068482

[B11] Ingale N. S. Motghare V. M. Gawade S. J. Sontakke S. D. Turankar A. V. (2021). Drug utilization study and adverse drug reactions profile of drugs in patients of ovarian cancer in tertiary care teaching hospital. Int. J. Pharm. Sci. Rev. Res. 66 (2), 74–79.

[B12] Ismail M. Khan S. Khan F. Noor S. Sajid H. Yar S. (2020). Prevalence and significance of potential drug-drug interactions among cancer patients receiving chemotherapy. BMC Cancer 20 (1), 335. 10.1186/s12885-020-06855-9 32307008 PMC7168989

[B13] Jayakumar A. Shekaina A. A. Kumar S. Chand S. George S. M. Joel J. J. (2021). Critical analysis of drug related problems among inpatients in the psychiatry department of a tertiary care teaching hospital: A pharmacist led initiative. Clin. Epidemiol. Glob. Health 11, 100743. 10.1016/j.cegh.2021.100743

[B14] Kamath A. Rai K. M. Shreyas R. Saxena P. U. P. Banerjee S. (2021). Effect of domperidone, ondansetron, olanzapine-containing antiemetic regimen on QTC interval in patients with malignancy: A prospective, observational, single-group, assessor-blinded study. Sci. Rep. 11 (1), 445. 10.1038/s41598-020-79380-1 33431995 PMC7801395

[B15] Kannan G. Anitha R. Rani V. N. Thennarasu P. Alosh J. Vasantha J. (2011). A study of drug-drug interactions in cancer patients of a south Indian tertiary care teaching hospital. J. Postgrad. Med. 57 (3), 206–210. 10.4103/0022-3859.85207 21941058

[B16] Key statistics for ovarian cancer (2022). Key statistics for ovarian cancer. Available from: https://www.cancer.org/cancer/ovarian-cancer/about/key-statistics.html (Accessed November 14th, 2022).

[B17] Kumar Y. E. P. Rajasekhar G. D. (2020). A study of prescription auditing in inpatient general medicine in tertiary care government hospital. Int. J. Res. Med. Sci. 8, 3979–3982. 10.18203/2320-6012.ijrms20204889

[B18] Lecours A. (2020). Scientific, professional and experiential validation of the model of preventive behaviours at work: Protocol of a modified Delphi study. BMJ Open 10, e035606. 10.1136/bmjopen-2019-035606 PMC7488793 32928848

[B19] Lee C. Y. Kim E. J. Hwang D. G. Jung M. Y. Cho H. G. (2019). The effect of trigger point injections on pain in patients with advanced cancer. Korean J. Fam. Med. 40 (5), 344–347. 10.4082/kjfm.18.0065 31487973 PMC6768837

[B20] MacKinnon N. J. Hartnell N. R. Black E. K. Dunbar P. Johnson J. Mahar S. H. (2008). Development of clinical indicators for type 2 diabetes. Can. Pharm. J. (Ott). 141 (2), 120–128. 10.3821/1913-701x(2008)141[120:docift]2.0.co;2

[B21] Mallik S. Palaian S. Ojha P. Mishra P. (2007). Pattern of adverse drug reactions due to cancer chemotherapy in a tertiary care teaching hospital in Nepal. Pak J. Pharm. Sci. 20 (3), 214–218. 17545106

[B22] Momenimovahed Z. Tiznobaik A. Taheri S. Salehiniya H. (2019). Ovarian cancer in the world: Epidemiology and risk factors. Int. J. Womens Health 11, 287–299. 10.2147/IJWH.S197604 31118829 PMC6500433

[B23] Mouhayar E. Salahudeen A. (2011). Hypertension in cancer patients. Tex Heart Inst. J. 38 (3), 263–265. 21720467 PMC3113122

[B24] National institute of cancer surveillance (2022). National institute of cancer surveillance. Available from: https://seer.cancer.gov/statfacts/html/ovary.html (Accessed November 14th, 2022).

[B25] Ni X. F. Yang C. S. Bai Y. M. Hu Z. X. Zhang L. L. (2021). Drug-related problems of patients in primary health care institutions: A systematic review. Front. Pharmacol. 2, 698907. 10.3389/fphar.2021.698907 PMC8418140 34489695

[B26] Oldak S. Ioannou S. Kamath P. Huang M. George S. Slomovitz B. (2019). Polypharmacy in patients with ovarian cancer. Oncologist 24 (9), 1201–1208. 10.1634/theoncologist.2018-0807 30952819 PMC6738286

[B27] Orkin A. M. Rao S. Venugopal J. Kithulegoda N. Wegier P. Ritchie S. D. (2021). Conceptual framework for task shifting and task sharing: An international Delphi study. Hum. Resour. Health 19 (1), 61. 10.1186/s12960-021-00605-z 33941191 PMC8091141

[B28] Poddar S. Sultana R. Sultana R. Akbor M. M. Azad M. A. K. Hasnat A. (2010). Pattern of adverse drug reactions due to cancer chemotherapy in tertiary care teaching hospital in Bangladesh. Dhaka Univ. J. Pharm. Sci. 8 (1), 11–16. 10.3329/dujps.v8i1.5330

[B29] Riechelmann R. Girardi D. (2016). Drug interactions in cancer patients: A hidden risk? J. Res. Pharm. Pract. 5 (2), 77–78. 10.4103/2279-042X.179560 27162799 PMC4843587

[B30] Ritchie M. K. Kohli A. (2022). Aprepitant. [Updated 2022 sep 22]. In: StatPearls [internet]. Treasure island (FL): StatPearls publishing; 2022 jan. Available from: https://www.ncbi.nlm.nih.gov/books/NBK551588/#_NBK551588_pubdet_ .

[B31] Sharma A. Ladd E. Unnikrishnan M. K. Kalra P. Gupta D. Sharma S. (2013). Healthcare inequity and physician scarcity: Empowering non-physician healthcare. Econ. Political Wkly. 48 (13), 112–119. 10.1016/j.jobcr.2013.08.001

[B32] Shrestha S. Shakya R. Shrestha S. Shakya S. (2017). Adverse drug reaction due to cancer chemotherapy and its financial burden in different hospitals of Nepal. Int. J. Pharmacovigil. 2 (1), 1–7. 10.15226/2476-2431/2/1/00114

[B33] Sisay E. A. Engidawork E. Yesuf T. A. Ketema E. B. (2015). Drug related problems in chemotherapy of cancer patients. J. Cancer Sci. Ther. 7 (2), 055–059. 10.4172/1948-5956.1000325

[B34] Taking multiple medicines safely (2023). Taking multiple medicines safely. [Updated 2023 February 22]. Available from: https://medlineplus.gov/ency/patientinstructions/000883.htm .

[B35] The Delphi method techniques and applications (2022). The Delphi method techniques and applications. Available from: https://web.njit.edu/∼turoff/pubs/delphibook/delphibook.pdf (Accessed November 14th, 2022).

[B36] Thiyagu R. Mallayasamy S. R. Rajesh V. Muralidhar V. Smitha P. Sudha V. (2010). Development of indicators for identifying adverse drug events in an Indian tertiary care teaching hospital. Drug Healthc. Patient Saf. 2, 95–100. 10.2147/dhps.s11222 21701622 PMC3108708

[B37] Treating ovarian cancer (2023). Treating ovarian cancer. Available from: https://www.cancer.org/cancer/ovarian-cancer/treating.html (Accessed February 23rd, 2023).

[B38] van Leeuwen R. W. F. Jansman F. G. A. van den Bemt P. M. L. A. de Man F. Piran F. Vincenten I. (2015). Drug-drug interactions in patients treated for cancer: A prospective study on clinical interventions. Ann. Oncol. 26 (5), 992–997. 10.1093/annonc/mdv029 25628444

[B39] van Roozendaal B. Krass I. (2009). Development of an evidence-based checklist for the detection of drug related problems in type 2 diabetes. Pharm. World Sci. 31 (5), 580–595. 10.1007/s11096-009-9312-1 19626455 PMC2730442

[B40] Wahlang J. B. Laishram P. D. Brahma D. K. Sarkar C. Lahon J. Nongkynrih B. S. (2017). Adverse drug reactions due to cancer chemotherapy in a tertiary care teaching hospital. Ther. Adv. Drug Saf. 8 (2), 61–66. 10.1177/2042098616672572 28255433 PMC5315222

[B41] What is ovarian cancer? (2022). What is ovarian cancer? Available from: https://www.cancer.org/cancer/ovarian-cancer/about/what-is-ovarian-cancer.html (Accessed November 14th, 2022).

[B42] Yeoh T. T. Tay X. Y. Si P. Chew L. (2015). Drug-related problems in elderly patients with cancer receiving outpatient chemotherapy. J. Geriatr. Oncol. 6 (4), 280–287. 10.1016/j.jgo.2015.05.001 26088749

[B43] Yokoyama S. Yajima S. Shimauchi A. Sakai C. Yamashita S. Noguchi Y. (2018). Oncology pharmacist contributions to treatment with oral anticancer agents in a Japanese community pharmacy setting. Can. Pharm. J. (Ott). 151 (6), 377–382. 10.1177/1715163518802865 30559912 PMC6293393

