# Supplementary material for: Development of evidence-based indicators for the detection of drug-related problems among ovarian cancer patients
Source: Front Pharmacol. 2023 Jun 30;14:1203648. doi: 10.3389/fphar.2023.1203648 (PMC10348894; doi:10.3389/fphar.2023.1203648)
Supplement: Supplementary file 1 [file Table1.docx]

| **A. Antineoplastic drugs**  Chemo therapeutic agents   - Vincristine - Vinblastine - Etoposide - Paclitaxel - Docetaxel - Doxorubicin - Bleomycin - Cyclophosphamide - Ifosfamide - Melphalan - Carboplatin - Cisplatin - Oxaliplatin - Gemcitabine - Capecitabine   Targeted drug therapy   - Bevacizumab - Olaparib - Niraparib - Rucaparib - Hormonal therapy - Tamoxifen   **B. Supportive therapy**  Cytoprotective   - Mesna   Antiemetic   - Aprepitant - Domperidone - Ondansetron - Palonosetron - Metoclopramide - Dexamethasone - Lorazepam   Antacids and gastroprotectant   - Pantoprazole - Rabeprazole - Ranitidine - Cimetidine - Famotidine - Mucaine gel (Oxetacaine + Aluminium Hydroxide + Milk of Magnesia) - Sucralfate | Anti-allergic   - Pheniramine - Hydrocortisone - Prednisolone - Levocetirizine - Chlorpheniramine   Analgesic and antipyretics:   - Paracetamol - Diclofenac - Naproxen, - Tramadol - Lorazepam - Pregabalin   Expectorant   - Bedyl: Guaifenesin+ Bromhexine+ Diphenhydramine+ Ammonium Chloride + Menthol) - Zerotuss: Ambroxol+ Levosalbutamol+ Guaifenesin - Asthmol-G: Ambroxol+ Levosalbutamol+ Guaifenesin   Dry cough   - Ascoril D (Phenylephrine+ Chlorpheniramine maleate+ Dextromethorphan hydrobromide)   Supplements   - Protein - Vitamins: B complex, Vitamin A, Vitamin E, Ascorbic acid, - Potassium and Mg: Kcl, MgSo4 - Calcium: Calcium+ vitaminD3 - Iron: ferrous   Laxative   - Lactulose - Milk of magnesia and liquid paraffin   Prophylactic anti-infections   - Augmentin - Cefixime - Metronidazole - Cefotaxime - Sulbactam   G-CSF   - Filgrastim - Pegfilgrastim   Anti-oxidants:   - Ascorbic acid - Ascorbic acid - Vitamin E   **Others:** Erythropoiesis stimulating agents (ESA), RBC transfusion |
| --- | --- |

**Supp. file 1: Drugs included in the indicators list**
