# Supplementary material for: Development of evidence-based indicators for the detection of drug-related problems among ovarian cancer patients
Source: Front Pharmacol. 2023 Jun 30;14:1203648. doi: 10.3389/fphar.2023.1203648 (PMC10348894; doi:10.3389/fphar.2023.1203648)
Supplement: Supplementary file 2 [file DataSheet1.docx]

Platinum Derivatives

Vinorelbine

CYP3A4 Inhibitors/ Inducer

Netupitant

Olaparib

COVID-19 Vaccine

G-CSF

**2.** Does the patient achieve these requirements:

If not, recommend

- **Age (>60 years):** Refer geriatric adult dose
- **BSA:** Calculate the dose based on the BSA
- **Stage of cancer**: Newly diagnosed, advanced, previously treated, relapsed

**Taxane derivatives**: **Docetaxel & Paclitaxel**

**CHEMOTHERAPY**

If Yes, continue and observe the possible ADRs

If No

Nausea and vomiting, diarrhea, stomatitis, flushing, ECG abnormality, edema, hypotension, peripheral neuropathy, arthralgia, myalgia, asthenia, alopecia and insomnia

Continue from section 8

- **Drug selection**: 1^st^ line drug to treat ovarian cancer is Paclitaxel in combination with Carboplatin
- **Route of administration:** IV

⮚**Previously treated**: IV: 135 or 175 mg/m^2^ over 3 hours every 3 weeks,

⮚**Previously untreated:** IV: 175 mg/m^2^ over 3 hours every 3 weeks (in combination with Cisplatin) or 135 mg/m^2^ over 24 hours administered every 3 weeks (in combination with cisplatin),

175 mg/ m^2^IV over 3 hours followed by Cisplatin 75 mg/ m^2^ every 3 weeks or 135 mg/ m^2^ IV over 24 hours followed by Cisplatin 75 mg/m^2^ every 3 weeks; premeditated with Dexamethasone 20 mg orally at approximately 12 and 6 hours before Paclitaxel plus Diphenhydramine 50 mg IV (or equivalent) and either Cimetidine 300 mg IV or ranitidine 50 mg IV 30 to 60 minutes before paclitaxel,

⮚135 mg/m^2^ or 175 mg/m^2^ IV over 3 hours every 3 weeks; premeditated with Dexamethasone 20 mg, orally at approximately 12 and 6 hours before Paclitaxel plus Diphenhydramine 50 mg IV (or equivalent) and either Cimetidine 300 mg IV or Ranitidine 50 mg IV 30 to 60 minutes before Paclitaxel

- **Drug preparation**: Vial should be reconstituted for infusion, it should be diluted in the NS 0.9, 5% Dextrose injections or 5% Dextrose injections, and 0.9% NS injections or Dextrose in ringers’ injections the final concentrations will be 0.3 to 12 mg/mL

If not, recommend

**4.** Does Paclitaxel administered concomitantly with:

Anticancer drugs may reduce the therapeutic activity of G-CSF

May alter the serum concentration of Paclitaxel

Myelosuppressive effect of Olaparib may enhanced

Therapeutic activity of vaccine may diminish

May enhance the adverse/toxic effect of Vinorelbine

May alter the serum concentration of Paclitaxel

Administer Paclitaxel before platinum derivatives

Monitor hematological and neurological parameters

Monitor the therapy

Administer 24 hour before or after the chemotherapy

**3.** Does the drug achieve these requirements:

**Supp. file 1: List of Indicators for Detection of DRPs in Ovarian Cancer Patients**

**1.** Does the patient Prescribed with Paclitaxel:

## 6. Lab Investigations:

## 5. Abnormal clinical changes in patient

Monitor the patient

- **Gastrointestinal:** Nausea and vomiting, diarrhea, stomatitis
- **Cardiovascular:** Flushing, ECG abnormality, edema, hypotension
- **Neurological:** Peripheral neuropathy
- **Hematological:** Anemia and fatigue
- **Renal:** Acute kidney injury, renal failure, dysuria, urinary incontinence, hematuria, CKD
- **Hepatic:** Hepatic function abnormal, liver injury, cholestasis, hepatic failure
- **Ophthalmic:** Eye infection
- **Respiratory:** Dyspnea, pneumonia
- **Dermatologic:** Skin rashes/alopecia, hypersensitivity reaction, changes in nails
- **Infections:** Sepsis
- **Others:** Injection site reaction, arthralgia, myalgia, asthenia
- **CBC:** Neutropenia, leukopenia, anemia, thrombocytopenia
- **RFT:** Proteinuria, raise in creatinine, urea
- **Electrolytes:** Electrolytes imbalance,
- **LFT:** Increased serum alkaline phosphatase, increased serum aspartate aminotransferase, increased serum bilirubin
- **Metabolic/ Endocrine changes:** Hypoalbuminemia, electrolytes imbalances
- **Imagination: CT scan, USG, X-ray** (tumor size reduction efficacy of drug/ other abnormalities

Monitor the patient

## 7. Patients care process Changes:

- **Withdrawal/ hold of medications:** Consider alternative chemotherapeutic agents for patients receiving or who have recently received amiodarone half-life of Amiodarone have t1/2 of 10-100 days. Hold the therapy until the neutrophil count reach >1,500/mm^3^ and platelets recover to 100,000/mm^3^. Reduce future doses by 20% for severe neutropenia (<500/mm^3^ for 7 days or more) and consider the use of supportive therapy, including growth factor treatment
- **Renal Impairment:** No specific dose recommendation for the renal disease but the drug should be given following the hemodialysis for the renal replacement therapy patients
- **Hepatic Impairment:** AST less than 2-10 times of upper limit Bilirubin <1,5mg/dl, 100mg/m^2^/ 24-hour infusion. Transaminase < 10 times of ULN, Bilirubin 1.6 to 7.5 mg/dL:50mg/m^2^ 24-hour infusion, >7.5mg/dl do not administer, transaminase levels less than 10 ULN, bilirubin levels of 1.26 to 2 times ULN 135 mg/ m^2^/ 3hr transaminase levels less than 10 times ULN and bilirubin levels of 2.01 to 5 times ULN, 90mg/ m^2^/3hr
- **ER visit/ Readmission to ICU/ Extend hospital stay**
- **Use of blood products**
- **Dose reduction:** For the potential interaction with drug CYP3A4 enzyme inhibitor and liver impairment case
- **Monitor:** Monitor for the effectiveness and toxicity of the drug when used together with strong inducer/ inhibitor of enzyme CYP3A4, neurotoxicity, blood parameters

Monitor the patient

If Yes, observe for the possible ADRs

If No

Nausea and vomiting, diarrhea, stomatitis, flushing, alopecia, ECG abnormality, edema, hypotension, peripheral neuropathy, arthralgia, myalgia, asthenia and fluid retention

Continue from section 16

**8.** Does the patient prescribed with Docetaxel:

**9.** Does the patient achieve these requirements:

- **Age (>60 years):** Adult dose refer
- **Weight, BSA, BMI:** If BMI more than >30mg/m^2^ then dose calculation more dose can require where BMI >30mg/m^2^ then dose reduction required
- **Stage of cancer:** Newly diagnosed, advanced, previously treated, relapsed

If not, recommend

**10.** Does the drug achieve these requirements:

If not, recommend

## Drug and dose based

- **Drug selection:** 1^st^ line drug to treat ovarian cancer in combination with Carboplatin

## Drug route and dose: IV: 60 mg/m^2^ every 3 weeks (in combination with Carboplatin) for up to 6 cycles or 75 mg/m^2^ every 3 weeks (in combination with carboplatin) for 6 cycles or 35 mg/m^2^ (maximum dose: 70 mg) weekly for 3 weeks followed by a 1-week rest (in combination with Carboplatin).

- **Drug administration/ use: IV:** Gently mixed the infusion with manual rotation. As this product is parenteral so should visually inspect and free from the any foreign particulates, precipitations, cristal, and discolorations in the solutions before administrations, if such issue observed then discards the solution from use.
- With maintaining the aseptic conditions withdraw the required amounts 20 mg/mL of Docetaxel from vials using 21-gauge needle**.** With a calibrated syringe and inject via a single injection (one shot) into a 250 mL infusion bag or bottle of either 0.9% NaCl or 5% Dextrose solution to produce a final concentration of 0.3 mg/mL to 0.74 mg/mL.
- Infuse IV through polyethylene-lined administration set over 1 hour under ambient temperature and lighting, bring vials to room temperature for about 5 minutes before preparation.
- **Note:** Observe the patients for 1^st^ few minutes for reactions if tolerated then go for infusion particularly1^st^ and 2^nd^ infusions. Slow IV administrations of infusion over the period of one hour. Whereas the premedication with the oral corticosteroids such as Dexamethasone 16 mg per day or 8 mg twice a day for 3 days stating 1 day prior to administration.

**11.** Does Docetaxel administered concomitantly with:

Platinum Derivatives

Vinorelbine

CYP3A4 Inhibitors/ Inducer

Netupitant

Olaparib

COVID-19 Vaccine

G-CSF

Antineoplastic agents may diminish the therapeutic effect of G-CSF

May alter the serum concentration of Docetaxel

Myelosuppressive effect of Olaparib may enhanced

Therapeutic activity of vaccine may diminish

May enhance the adverse/toxic effect of Vinorelbine

May alter the serum concentration of Docetaxel

Administer Taxel before platinum derivatives

Monitor hematological and neurological parameter

Monitor the therapy

Administer 24 hour before or after the chemotherapy

**12.** Check if this formulation is contraindicated to patient:

Neutrophil counts of less than 1500 cells/mm^3^, baseline neutrophil counts <1,000/mm^3^, hypersensitivity to Paclitaxel

Avoid use

## 13. Abnormal clinical changes in patient

- **Gastrointestinal:** Nausea and vomiting, diarrhea, stomatitis, dysgeusia
- **Cardiovascular:** Sinus tachycardia, syncope, tachycardia, hypotension
- **Neurological:** Peripheral neuropathy, dysesthesia, paresthesia
- **Hematologic:** Paleness, fatigue, breathlessness, infection
- **Renal:** Acute kidney injury, renal failure, dysuria, haematuria, urinary retention
- **Hepatic:** Hepatic function abnormal, liver injury, hepatic failure, cholelithiasis, hepatitis
- **Ophthalmic:** Eye infection
- **Respiratory:** pulmonary disease, pulmonary embolism, difficulty in breathing,
- **Dermatologic:** Skin rashes, alopecia, hypersensitivity reaction,
- **Infections:** Infection, sepsis
- **Others:** Injection site reaction, arthralgia, myalgia, asthenia, fluid retention, tumor lysis syndrome

Monitor the patient

## 14. Lab Investigations:

Monitor the patient

- - **CBC:** Neutropenia, leukopenia, anemia, thrombocytopenia

## RFT: Raised serum creatinine, proteinuria

- - **Electrolytes:** Electrolyte imbalance
  - **LFT:** Increased serum alanine aminotransferase, increased aspartate aminotransferase
  - **Metabolic/ Endocrine changes:** Electrolyte disorder, hypocalcemia, hypokalemia, hypomagnesemia, hyponatremia
  - **Imagination: CT scan, USG, X-ray** (tumor size reduction efficacy of drug/ other abnormalities)

## 15. Patients care process Changes:

:

- - **Sudden withdrawn/ hold of medications:**
  - **Renal Impairment:** Dose adjustment may require in reduced renal excretions
  - **Hepatic Impairment:** If bilirubin level >ULN or Transaminase level 1.5 times of UNL and or ALP > by 2.5 times of UNL then avoid use of docetaxel
  - **ER visit/ Readmission to ICU/Extend hospital stay**
  - **Use of blood products**
  - **Dose reductions:** For the potential interaction with drug CYP3A4 enzyme inhibitor and liver impairment case
  - **Monitor:** Monitor for the effectiveness and toxicity of the drug when used together with strong inducer/ inhibitor of enzyme CYP3A4, neurotoxicity, blood parameters, prevent intoxications of alcohol use is contraindicated in the patients, reactions cutaneous, tumor lysis syndrome

Monitor the patient

If Yes, Observe the possible ADRs

If No

Decrease in WBC or platelet counts, cause severe eye irritation or corneal ulceration, serum uric acid, fetal harm, shortness of breath, progressive dyspnea abnormal variations in heart rate and blood pressure, hair loss, constipation, nausea and vomiting, cranial nerve disorder, neurotoxicity, paralysis, seizure, vocal cord paralysis

Continue from section 24

**16.** Does the patient prescribed with Vincristine:

**Vinca alkaloids:Vincristine &Vinorelbine**

**17.** Does the patient achieve these requirements:

- **Age (>60 years):** Adult dose refer
- **BSA, BMI:** Obese adult can be given maximum 2mg
- **Type of cancer:** Germ cell tumor of ovary

If not, recommend

**18.** Does the drug achieve these requirements:

If not, recommend

## Drug and dose based

- **Drug selection:** Drug of choice for germ cell tumor, VAP, VIP,
- **Route and dose**: **IV:** VAC regimen: 1.5 mg/m^2^ (maximum dose: 2 mg) once weekly for 8 to 12 weeks (in combination with Dactinomycin and Cyclophosphamide, Taxel)
- **Drug preparation:** Obtain the concentration of Vincristine 1mg/ml. Avoid addition of extra fluids in the vials before withdrawal of dose. Take out the accurate quantity of dose into a dry syringe. Similarly, do not put the extra solvent in the vials for the purpose of entirely empty the vials. 0.9% NS, D5W can be used as diluents.
- **Note:** Only for IV use. Don’t administer by intrathecally warning should be given in the labeling
- **Drug dispense:** under dose, over dose, wrong dosage form, wrong drug, dispense Vincristine in a mini-bag or other flexible plastic container (NOT in a syringe**)**
- **Drug administration/ use:** IV Inspect visually and make ensure that product is free from the visual particles and discolorations before use**.** Short 5to 10min infusion free flowing IV line near the mini bag followed by the flushing with minimum 25 to 50 mL 0.9%NS or D5W. Some case 24-hour infusion rate, or slow push 1-2 min. Vincristine should not be delivered to the patient at the same time with any medications intended for CNS administration. Ensure regarding the proper catheter placed and avoid the extravasations

**19.** Does Vincristine prescribed concomitantly with:

Nifedipine

CYP3A4 Inhibitors/ Inducer

Netupitant

Digoxin

G-CSF

Vincristine and G-CSF used together may leads to the peripheral neuropathy

May increase the risk of Vincristine toxicity

May result in decreased digoxin effectiveness

May alter the serum concentration of Vincristine

Monitor patients for neuropathy, delirium, seizures

Monitor the therapy

Restrict the total dose of Vincristine used in the first cycle and monitor patients

**20.** Check if this formulation is contraindicated to patient:

Severe allergic reactions to the Vincristine or similar chemical structure compounds, and demyelinating form of Charcot-Marie-Tooth Syndrome patients.

Avoid use

**21.** Abnormal clinical changes in patient:

Monitor the patient

- **Gastrointestinal:** Nausea and vomiting, abdominal cramps, anorexia, constipation, diarrhea, intestinal necrosis, intestinal perforation, nausea, oral mucosa ulcer, paralytic ileus
- **Cardiovascular:** Hypotension, hypertension
- **Neurological:** Peripheral neuropathy, sensory loss, paralysis, seizure
- **Hematologic:** Leukopenia
- **Renal:** Bladder dysfunction, dysuria, polyurea
- **Hepatic:** Veno-occlusive disorder of liver**,** portal hepatitis, hepatomegaly
- **Ophthalmic:** Blepharoptosis, cortical blindness
- **Respiratory:** acute respiratory distress syndrome, dyspnoea, cough, epistaxis, tachypnea
- **Dermatologic:** Skin rashes / alopecia,changes in nail
- **Infections:** Risk of infections
- **Others:** Weight loss, fever

## 22. Lab Investigations:

Monitor the patient

- **CBC:** Leukopenia, neutropenia,

## RFT: Protein urea

- **Electrolytes:** Electrolyte imbalance
- **LFT:** Elevated bilirubin, transaminase
- **Metabolic/ Endocrine changes:** Hyperuricemia, metabolic acidosis, increase blood glucose
- **Imagination: CT scan, USG, X-ray** (tumor size reduction efficacy of drug/ other abnormalities)

## 23. Patients care process Changes:

- **Sudden withdrawn/ hold of medications:**
- **Renal Impairment:** No need to adjust dose.
- **Hepatic Impairment:** Serum bilirubin 1.5 to 3 mg/dL or transaminases 2 to 3 times ULN or alkaline phosphatase increased: Administer 50% of dose. Serum bilirubin 1.5 to 3mg/dL: Administer 50% of dose, Serum bilirubin >3 mg/dL: Avoid use.

## ER visit/ Readmission to ICU/ Extend hospital stay

- **Use of blood products**
- **Dose reductions:** Serum bilirubin 1.5 to 3 mg/dL: Administer 50% of dose, Serum bilirubin >3 mg/dL: Avoid use.
- **Monitor:** Monitor for the effectiveness and toxicity of the drug when used together with
- Strong inducer/ inhibitor of enzyme CYP3A4.
- **Drug to be avoid use together:** CNS acting drug

Monitor the patient

Paclitaxel

Cisplatin

CYP3A4 Inhibitors/ Inducer

Netupitant

Olaparib

COVID-19 Vaccine

G-CSF

If Yes, observe for possible ADRs

If No

Decrease in WBC or platelet counts, cause severe eye irritation or corneal ulceration, serum uric acid, fetal harm, shortness of breath, progressive dyspnea, abnormal variations in heart rate and blood pressure, hair loss, constipation, nausea and vomiting, cranial nerve disorder, neurotoxicity, paralysis, seizure, vocal cord paralysis

Continue from section 32

**24.** Does the patient prescribed with Vinorelbine:

**25.** Does the patient achieve these requirements

- **Age (>60 years):** Refer normal adult dose
- **BSA, BMI:** Obese adult can be calculated based on the body weight and the BSA
- **Types and stage of cancer:** Advanced, previously treated, relapsed ovarian cancer

If not, recommend

**27.** Check if this formulation is contraindicated to patient:

Hypersensitivity to vinorelbine or any component of the formulation; drug-induced severe granulocytopenia or severe thrombocytopenia.

Avoid use

**26.** Does the drug achieve these requirements:

If not, recommend

## Drug and dose based

- **Drug selection:** Relapsed ovarian cancer
- **Route and dose**: **IV:** 25 to 30 mg/m^2^ weekly or 30 mg/m^2^ day 1 and 8 in the treatment cycle of 21 days treatment cycle, till the condition progresses or intolerable toxicity
- **Note:** Should not contain the preservative
- **Drug preparation:** Vinorelbine injection can be diluted in the IV bag or syringe with suitable solutions. The calculated dose of Vinorelbine in syringe can be 1.5 to 3mg/ml in 5% Dextrose or 0.9% NS diluents, whereas in Infusion bag the calculated dose of the drug can be from 0.5 to 2mg/ml in the injections; 5% Dextrose or 0.9% NS or 0.45% sodium chloride, ringer, 0.45% sodium chloride and 5% dextrose diluents.
- **Drug administration/ use: IV:** Only for IV route, other routes could be lethal. Administer within 6 to 10 minutes through

side port of the freely flowing IV line. To prevent incidence of phlebitis infused 75 to 125ml of compatible solution following the drug administration

**28.** Does Vinorelbine prescribed concomitantly with:

Antineoplastic agents may diminish the therapeutic effect of G-CSF

May alter the serum concentration of Paclitaxel

Myelosuppressive effect of Olaparib may enhanced

May diminish the therapeutic effect of COVID-19 Vaccine

May enhance the adverse/toxic effect of Vinorelbine

May alter the serum concentration of Paclitaxel

Monitor for hematological toxicity

Monitor for granulocytopenia

Monitor the therapy

Administer 24 hour before or after the chemotherapy

## 29. Abnormal clinical changes in patient:

- **Gastrointestinal:** Nausea and vomiting, anorexia, constipation, diarrhea
- **Cardiovascular:** Localized phlebitis, chest pain
- **Neurological:** Peripheral neuropathy, sensory loss, paralysis, seizure
- **Hematologic:** Anemic, susceptible for infection (weaken immune system)
- **Renal:** Acute kidney injury, renal failure
- **Hepatic:** Hepatic function abnormal, hepatic failure
- **Ophthalmic:** Visual impairment**, c**ataract, blurred vision
- **Respiratory:** Dyspne**a**
- **Dermatologic:** Skin rashes / alopecia
- **Infections:** Sepsis
- **Others:** Injection site reaction, ototoxicity

Monitor the patient

## 30. Lab Investigations:

Monitor the patient

- **CBC:** Neutropenia, leukopenia, anemia, thrombocytopenia
- **RFT:** Increased serum creatinine
- **LFT:** Increased serum aspartate aminotransferase
- **Metabolic/Endocrine changes:** Decreased appetite, dehydration, hypokalemia, hyponatremia, hyperglycemia
- **Imagination: CT scan, USG, X-ray** (tumor size reduction efficacy of drug/ other abnormalities**)**
- **Sudden withdraw/ hold of medications:**
- **Renal Impairment:** No need to adjust dose for renal disease but reduce the dose up to 20mg/m^2^ for hemodialysis patients, administer drug after dialysis or non-dialysis.
- **Hepatic Impairment:** Serum bilirubin ≤2 mg/dl can be given the normal dose, where the serum bilirubin in 2.1 to 3mg/dl then can be given 50% of normal dose, S. bilirubin greater than 3 mg/dL then 25% of the normal dose should be given.
- **ER visit/ Readmission to ICU/ Extend hospital stay**
- **Use of blood products**
- **Dose reductions:** Hemodialysis, hepatic impairments
- **Monitor:** Monitor for the effectiveness and toxicity of the drug when used together with strong inducer/ inhibitor of enzyme CYP3A4
- **Drug to be avoid use together:** CNS acting drug

Monitor the patient

## 31. Patients care process Changes:

:

If Yes, observe for the possible ADRs

If No

Tumor pain, hyperpigmentation, dermatologic: phlebitis, hyperpigmentation, atrophic striae, erythema, exfoliation of the skin on the palmar and plantar surfaces of the hands and feet), hyperkeratosis, localized vesiculation, skin rash, skin sclerosis, alopecia, nailbed changes

Continue from section 40

**32.** Does the patients prescribed Bleomycin:

**Antitumor antibiotics: Doxorubicin & Bleomycin**

**33.** Does the patient achieve these requirements:

- **Age (>60 years)**: Refer normal adult dose, but higher incidence of pulmonary toxicity in >70 years age.
- **BSA, BMI**: Dose calculation according to the BSA, and consider when hepatic and renal impairment occurs.
- **Types and stage of cancer**: Germ cell tumor of ovary

If not recommend

**34.** Does the drug achieve these requirements:

If not, recommend

## Drug and dose based

- **Drug selection:** BEP regimen on the successful combination for treatment of germ cell tumor
- **Route and dose**: **IV:** BEP regimen: IV: 30 units/dose days 1, 8, and 15 of a 21-day treatment cycle for 3 cycles (in combination with Etoposide and Cisplatin) **or** 15 units/m^2^ day 1 of a 21-day treatment cycle for 4 cycles (in combination with Etoposide and Cisplatin), maximum dose not more than 400 units’ total dose
- **Drug preparation**: It is recommended that Bleomycin to be administered under the supervision of a qualified physician experienced in the use of cancer chemotherapeutic agents. This parenteral drug products should be inspected visually and make ensure that free from the foreign particles and discolorations before administrations

## Drug administration/ use: IV:

## IV doses should be administered slowly over 10 minutes (according to the manufacturer's labeling), the drug will be administered in Day 1, 8 and 15of 3-4 weeks each cycle

Hypersensitivity to vinorelbine or drug-induced severe granulocytopenia or severe thrombocytopenia.

Avoid use

**35.** Check if this formulation is contraindicated to patient:

## 37. Abnormal clinical changes in patient:

- **Gastrointestinal:** Stomatitis, mucositis, anorexia
- **Cardiovascular:** Atrioventricular block, bradycardia, bundle branch block, ECG abnormality, extrasystoles (atrial or ventricular), nonspecific ST or T wave changes on ECG, sinus tachycardia, supraventricular tachycardia, tachyarrhythmia, ventricular tachycardia, cardiac failure delayed
- **Neurological and /SKM Effects:** Cerebral arteritis, combination with cisplatin and vinca-alkaloids  encephalopathy, seizure, cerebral **edema and/** malaise, weakness
- **Hematologic:** Pale skin, fatigue, cyanosis
- **Renal:** Acute kidney injury, dysuria, anuria, oliguria, burning micturition, hematuria
- **Hepatic:** Hepatitis, veno-occlusive disease of the liver, jaundice, acute hepatic failure, hepatomegaly
- **Ophthalmic:** Discoloration of tears, eye infections
- **Respiratory:** Pulmonary pneumonitis, pulmonary fibrosis, pulmonary toxicity increases by radiations therapy
- **Dermatologic:** Hyperpigmentation, atrophic striae, erythema, exfoliation of the skin on the palmar and plantar surfaces of the hands and feet, hyperkeratosis, localized vesiculation, skin rash, skin sclerosis, alopecia, nailbed changes
- **Infections:** Pneumonia, sepsis, UTI, fungal infections
- **Others:** Injection site reaction, weight loss, decreased appetite, dehydration

Monitor the patient

**36.** Does the Bleomycin concomitantly prescribed with:

Phenytoin

Gemcitabine

Oxygen

G-CSF

Ascorbic acid

May increase the risk for pulmonary toxicity of Bleomycin

May reduce the serum concentration of Phenytoin

Monitor the therapy

Administer 24 hour before or after the chemotherapy

Keep O_2_ at room air, and monitor the patient for pulmonary toxicity

May diminish the efficacy of Bleomycin

If interaction suspected discontinue the Ascorbic acid

## 38. Lab Investigations:

Monitor the patient

- - **CBC:** Neutropenia, leukopenia, anemia, thrombocytopenia
  - **RFT:** CrCL
  - **Electrolytes:** Electrolytes imbalances
  - **LFT:** Transaminase level increases, bilirubin level increases
  - **Metabolic and endocrine disorder:** Secretions of antidiuretic hormone inappropriately,
  - **Imagination: CT scan, USG, X-ray** (tumor size reduction efficacy of drug/ other abnormalities**)**

## 39. Patients care process Changes:

- - **Sudden withdrawn/ hold of medications**:
  - **Renal Impairment:** RFT check, dosing calculation required when CrCl clearance reduced
  - **Hepatic Impairment:** No dosage adjustment required
  - **ER visit/ Readmission to ICU/ Extend hospital stay**
  - **Use of blood products**
  - **Dose reductions:** Renal impairment conditions
  - **Monitor:** Pulmonary function tests, renal function, liver function, for therapy efficacy: tumor response,

Monitor the patient

If Yes, Observe for possible ADRs

If No

Alopecia, GI issue, cardiomyopathy, congestive heart failure, left ventricular failure, acute, myocardial infarction, myocarditis, pericarditis, tachyarrhythmia, leukopenia, neutropenia, thrombocytopenia, hepatitis, anaphylaxis, septic shock, tumor lysis syndrome, discoloration of tears

Continue from section 48

**40.** Does the patient prescribed with Doxorubicin:

**41.** Doe**s** the patient achieve these requirements

- **Age (>60 age):** Refer lower dose than the recommended, and maintain more gaps between the two cycles
- **BSA, BMI:** Dose calculation according to BSA, BMI, and consider when hepatic and renal impairment occurs.
- **Types and stage of cancer:** Ovarian cancer (metastatic), Epithelial ovarian cancer

If not, recommend

**42.** Does the drug achieve these requirements:

If not, recommend

## Drug and dose based

- **Drug selection:** Drug of choice for the metastatic ovarian cancer
- **Route and dose**: **IV:** Single 60 to 75 mg/m^2^ IV every 21 days, 40 to 75 mg/m^2^ IV every 21 to 28 days,
- **Drug preparation**: The sterile lyophilized powder can be diluted with 0.9%NS, D5W results the final concentrations of 2mg/ml. The IV Push syringe have concentrations of 2mg/ml pushed over the 5 to 10minutes, whereas the IV infusion have the concentrations of 0.2mg-2mg/ml, volume of 50-1000ml infused over the 30 to 60 minutes

## Drug administration/ use: IV: Administer into a central line/ main vein or secure and freely running peripheral IV of 0.9% or 0.45% sodium chloride or D5W, IV push over 3 to 10 minutes; decrease rate if erythematous streaking along the vein or facial flushing occurs. Infusion will be administered over the time period of 30 to 60 minutes.

- **Continuous infusion**: Infuse only through a central catheter; decrease rate if erythematous streaking along the vein or facial flushing occurs
- Administer Doxorubicin hydrochloride prior to paclitaxel if used concomitantly
- **Note:** Dose administration time should not be less than the 3-5 min, indicators for the rapid push are erythematous steaking along with veins, facial flushing, whereas burning and tingling sensations may be due to the perivenous injections then immediately removed the injections and restart in another vein. Should not be given in IM or SC route, discard the unused drug from the single dose vial similarly discard the unused drug dose from the multiple beyond the recommended storage time period.

Drug-induced, persistent, severe myelosuppression, recent myocardial infarction occurring within the past 4 to 6 weeks, severe myocardial insufficiency, severe hypersensitivity reaction to Doxorubicin hydrochloride, Anthracyclines with Bevacizumab concomitant use

Avoid use

**43.** Check if this formulation is contraindicated to patient:

**44.** Does the Doxorubicin concomitantly prescribed with:

Bevacizumab

Cardiac Glycosides

Olaparib

Cyclophosphamide

CYP3A4 inhibitors

G-CSF

COVID-19 Vaccine

May decrease the serum concentration of cardiac glycosides

May enhance the cardiotoxic effect of conventional Doxorubicin

Avoid combination

Antineoplastic agents may diminish the therapeutic effect of G-CSF

Monitor therapy

Myelosuppressive agents may enhance the myelosuppressive effect of Olaparib

Administer 24 hour before or after the chemotherapy

May enhance the cardiotoxic effect of anthracyclines

May result in increased Doxorubicin exposure

If interaction suspected the discontinue the Ascorbic acid

Antineoplastic agents may diminish the therapeutic effect of G-CSF

Monitor the therapy

## 45. Abnormal clinical changes in patient

- **Gastrointestinal:** Abdominal pain, anorexia, diarrhea, discoloration of saliva, gastrointestinal ulcer, mucositis, nausea, vomiting, pancreatitis
- **Cardiovascular:** Atrioventricular block, bradycardia, bundle branch block, ECG abnormality, extrasystoles (atrial or ventricular), nonspecific ST or T wave changes on ECG, sinus tachycardia, supraventricular tachycardia, tachyarrhythmia, ventricular tachycardia, cardiac failure delayed
- **Neurological:** Malaise, weakness
- **Hematologic:** looks anemia and fatigue
- **Renal:** Renal failure, dysuria, hematuria
- **Hepatic:** Hepatitis, veno-occlusive disease of the liver, jaundice
- **Ophthalmic:** Discoloration of tears, eye infections, lacrimation increased, vision blurred, visual disturbance, blindness, eye pain, edema of eye lid
- **Respiratory:** Dyspnea, pleural effusions, cough, oropharyngeal pain, pulmonary oedema, acute respiratory distress syndrome, bronchospasm, epistaxis
- **Dermatologic:** Alopecia, discoloration of sweat, pruritus, skin photosensitivity, skin rash, urticaria, changes in nail
- **Others:** Injection site reaction, arthralgia, myalgia, asthenia, amenorrhea, dehydration, urine discoloration, infertility, infection

Monitor the patient

## 46. Lab Investigations:

Monitor the patient

- **CBC:** Neutropenia, leukopenia, anemia, thrombocytopenia
- **RFT:** Abnormal renal parameter may observe
- **Electrolytes:** Hypokalemia, hyperglycemia, hyponatremia, hypocalcemia
- **LFT:** Increase bilirubin level of blood, AST, ALT, ALP
- **Metabolic/ Endocrine changes:** hyperuricemia, electrolytes imbalance
- **Imagination: CT scan, USG, X-ray** (tumor size reduction efficacy of drug/ other abnormalities**)**

## 47. Patients care process Change:

- - **Sudden withdraw/ hold of medications**: Cardiomyopathy**:** Discontinue therapy if signs or symptoms develop, avoid the drug use if serum bilirubin 5mg/dl or above
  - **Renal Impairment:** Normal dose can be given, hemodialysis give normal dose after dialysis.

Monitor the patient

## 47. Change in Patients care process:

- - **Hepatic Impairment:** Serum bilirubin 3.1 to 5 mg/dl reduce dose by 75%, and 1.2 to 3 mg bilirubin reduce dose by 50% and avoid use if 5mg/dl if S. bilirubin
  - **ER visit/** **Readmission to ICU/Extend hospital stay**
  - **Use of blood products**
  - **Dose reductions:** Hepatic impairment may require
  - **Monitor:** Monitor for the effectiveness and toxicity of the drug when used together with strong inducer/ inhibitor of enzyme CYP3A4, neurotoxicity, blood parameters, hepatic parameters, cardiac monitoring, ECG, electrolytes
  - **Drug to be avoid use together:** Moderate and strong inhibitors or inducer of CYP3A4 of CYP2D6

Monitor the patient

If Yes, observe for the possible ADRs

If No

Alopecia, nausea and vomiting, anorexia, diarrhea, leukopenia, thrombocytopenia, anemia, hypotension, hypersensitivity, tachycardia, dyspnea, peripheral neuropathy, abdominal pain, hepatotoxicity

Continue from section 56

**48.** Does the patient prescribed with Etoposide:

**Epipodophyllotoxins**: **Etoposide**

**49.** Does the patient achieve these requirements:

- **Age (>60 years):** Refer adult dose
- **BSA, BMI:** Dose calculation according to the BSA, and consider when hepatic and renal impairment occurs.
- **Types and stage of cancer**: Germ cell tumor of ovary, and platinum-resistant/refractory epithelial ovarian cancer

If not, recommend

**50.** Does the drug achieve these requirements:

If not, recommend

## Drug and dose based

- **Drug selection:** BEP regimen on the successful combination for treatment of germ cell tumor
- **Route and dose**: **IV: Ovarian cancer, epithelial, refractory**: Oral: 50 mg/m2 OD for 21 days every 4 weeks until disease progression or unacceptable toxicity.
- **Ovarian germ cell tumors: BEP regimen: IV:**100 mg/m^2^ on days 1 to 5 every 21 days (in combination with bleomycin and cisplatin) for 3 cycles
- ***EP regimen****:* **IV:**100 mg/m^2^ on days 1 to 5 every 21 days (in combination with cisplatin) for 4 cycles; while the BEP regimen is preferred in the treatment of ovarian germ cell tumors, EP may be considered if pulmonary toxicity is a concern. **Note:** Use of this regimen in ovarian germ cell
- **Carboplatin/etoposide regimen***:* **IV:**120 mg/m^2^ days 1, 2, and 3 every 4 weeks (in combination with carboplatin) for 3 cycles
- **Preparation:** Dilute to the 5% of dextrose or normal saline, obtain final concentration 0.2 to 0.4mg/ml. The concentration more than 0.4 mg/dl may occur precipitation.

## Drug administration/ use: IV: Rapid infusion may lead to the hypotension so infuse over the time period of 30 to 60 minutes. A duration of infusion will be adopted if volume of fluid is required. Avoid rapid infusion of Etoposide

**51.** Does the Etoposide concomitantly prescribed with:

Olaparib

CYP3A4 Inducers (moderate)

CYP3A4 Inducers (strong)

G-CSF

May decrease the serum concentration of Etoposide

May enhance the myelosuppressive effect of Olaparib

Monitor the therapy

Administer 24 hour before or after the chemotherapy

Increase the dose of Etoposide

May diminish the therapeutic effect of Lenograstim

Hypersensitivity to Etoposide or any component of the formulation

Avoid use

**52.** Check if this formulation is contraindicated to patient:

**53**. Abnormal clinical changes in patient:

Monitor the patient

- **Gastrointestinal:** Anorexia, diarrhea, nausea, vomiting, metallic taste, mouth sore
- **Cardiovascular:** Hypotension on rapid infusion, palpation, light headache
- **Neurological:** Seizure, numbness, tingling effect
- **Hematologic:** Pale skin, fatigue, risk of bleeding
- **Renal:** Renal failure, dysuria, hematuria
- **Hepatic:** Hepatitis, veno-occlusive disease of the liver, hepatomegaly
- **Ophthalmic:** Vision blurred, visual disturbance, blindness
- **Respiratory:** Dyspnea, cough, acute respiratory distress syndrome, pleural effusion, pneumonitis, bronchospasm, hiccups
- **Dermatologic:** Alopecia, discoloration of sweat, pruritus, maculopapular skin rash, urticaria, erythematous rashes
- **Others:** Injection site reaction, arthralgia, myalgia, asthenia, amenorrhea, dehydration, urine discoloration, infertility, fever, decrease albumin level, weak immune system

**54**. Lab Investigation

Monitor the patient

- **CBC:** Leukopenia, anemia, thrombocytopenia
- **RFT:** Abnormal renal parameter may observe
- **LFT:** Increase bilirubin level of blood, AST, ALT, ALP
- **Metabolic/ Endocrine changes:** hyperuricemia, electrolytes imbalance, T_3_ and T_4_ level low, TSH level high
- **Imagination: CT scan, USG, X-ray** (tumor size reduction efficacy of drug/ other abnormalities**)**

**55**. Patient care process change:

If not, recommend

- **Sudden withdrawn/ hold of medications**: Neutrophil <500/mm^3^ or platelets <50,000/mm^3^ until recovery, hypersensitive reaction or severe ADRs.
- **Renal Impairment:** If the GFR>50ml /min then administer 75% of the total dose and if less than 10ml/min GFR then administer 50% of the dose. For dialysis patients reduce 50% to 75% of the dose and administer after the dialysi
- **Hepatic Impairment:** Administer 50% of the dose if bilirubin elevated to 1.5- 3 mg/dL or AST >3 times ULN:
- **ER visit/** **Readmission to ICU/ Extend hospital stay**
- **Use of blood products**
- **Dose reductions:** Hepatic impairment may require
- **Monitor:** RFT, LFT, infusion reaction, blood pressure, CBC, tumor response

Alopecia, nausea and vomiting, anorexia, diarrhea, leukopenia, thrombocytopenia, anemia, hypotension, hypersensitivity, tachycardia, dyspnea, peripheral neuropathy, abdominal pain, hepatotoxicity, altrered sense of smell

**56.** Does the patient prescribed with Cyclophosphamide:

If Yes, observe the possible ADRs

If No

Go the section 64

**Nitrogen mustard: Cyclophosphamide, Ifosfamide & Melphalan**

- - **Age (>60 years)**: Refer adult dose
  - **BSA:** Calculate the dose accordance to the BSA, adjust renal clearance
  - **Types and stage of cancer**: Ovarian germ cell tumors (ovarian adenocarcinoma), malignant

If not, recommend

**57.** Does the patient achieve these requirements:

**58**. Does the drug achieve these requirements:

If not, recommend

## Drug and dose based

- **Drug selection:** Drug of choice for adenocarcinoma of ovary
- **Route and dose**: 150 mg/m^2^ IV on day 1 to day 5 repeat every 28 days, minimum of 10 cycles in combination with Dactinomycin and Vincristine (VAC).
- IV 600mg/m^2^ in combination with Carboplatin 300mg/m2 in day 1, repeat every 4 week for 6 cycles.
- **Single:**  40 to 50 mg/kg IV in divided doses over 2 to 5 days OR 10 to 15 mg/kg IV every 7 to 10 days OR 3 to 5 mg/kg IV twice weekly or **Oral:** Cyclophosphamide is usually administered at dosages in the range of 1 to 5 mg/kg/day for both initial and maintenance dosing.
- **Drug preparation:** Reconstitute the drug with aim to make 20mg/ml in NS/DNS but not in WFI**. Reconstituted** solution should be diluted in 5DW/5D/NS to obtain minimum of 2mg/ml. Agitate the vials vigorously for dissolving the drug in the solvent.
- **Drug administration/ use**: Before using the product make ensure that product should be inspect visually and most free from any visible particulates, discolorations, and also solutions and container should meet the criteria.
- **Note:** The drug constituted in water should not be administer directly due to the hypotonic nature.

Hypersensitivity to Cyclophosphamide or cross reaction with alkylating agents

Avoid use

**59.** Check if this formulation is contraindicated to patient:

**60.** Does Cyclophosphamide concomitantly prescribed with:

Anthracyclines

Olaparib

Amiodarone

Ondansetron

Glimepiride

Netupitant

Thiazide-Like Diuretics

CYP3A4 (Inducers or inhibitor)

Digoxin

G-CSF

May enhance the myelosuppressive effect of Olaparib

May enhance the cardiotoxic effect of Anthracyclines

Monitor the therapy

Administer 24 hour before or after the chemotherapy

May result in decreased Digoxin serum concentration

May escalate the risk of pulmonary toxicity of Cyclophosphamide and even immunosuppressant may reduce the effectiveness of the G-CSF

Substitute digoxin in liquid form or capsule

Netupitant may result in reduced Cyclophosphamide efficacy.

May alter the serum concentration of Cyclophosphamide

May enhance toxic effects of Cyclophosphamide mainly granulocytopenia may be enhanced

May decrease Cyclophosphamide systemic exposure

May enhance the risk of pulmonary toxicity

May increase blood glucose lowering effect and risk of hypoglycemia

Monitor the therapy for efficacy and adverse effects

## 61. Abnormal clinical changes in patient:

- **Gastrointestinal:** Abdominal pain, anorexia, diarrhea, mucositis, stomatitis, nausea, vomiting
- **Renal:** Acute urinary retention, urinary incontenance
- **Hepatic:** Hepatic encephalopathy, altered sensorium, hepatomegaly**,** jaundice

Monitor the patient

## 62. Lab Investigations:

## 61. Abnormal clinical changes in patient:

Monitor the patient

- **Respiratory:** Dyspnea, breathing problems

## Dermatologic: Skin rashes /angioedema/SJS/ alopecia, changes in nails

## Infections: Severe infection, (requires hospitalization, IV antibiotics)

- **Others:** Facial swellings rapid infusion time, hemorrhagic cystitis
- **CBC:** Observe neutrophil: 1500/mm^3^ or lower and platelets: <50,000/mm^3^, avoid use. Monitor CBC and dose adjustment or terminate the drug
- **RFT:** S. Creatinine and urea level monitor dose adjustment or terminate the drug. Monitor S. creatinine when fluconazole used together.
- **Electrolytes:** Hyponatremia
- **LFT:** Serum bilirubin 3.1 to 5 mg/dL or transaminases >3 times ULN: Administer 75% of dose, if S. bilirubin< 5mg/dl avoid use, monitor bilirubin when Fluconazole used together
- **Imagination: CT scan, USG, X-ray** (tumor size reduction efficacy of drug/ other abnormalities)

Monitor the patient

## 63. Patients care process Changes:

- **Sudden withdraw of medications:** Hemorrhagic cystitis severe
- **Nausea/ vomiting**: Moderate to high emetic drug, antiemetic recommended
- **ER visit/ Readmission to ICU/ Extend hosptal stay**
- **Use of blood products**
- **Dose reductions:** Dose reductions or interruptions of drug may require hematologic toxicity,
- **Monitor:** Monitor the tumor response and clinical evidence for the therapy efficacy, CBC, cardiac toxicity for patients having history of cardiac disease, renal impairments parameter**s**
- **Use of renal protectant (diuretics, hydration):** Co-administer with adequate amounts of fluids during and within 1 to days, with or without Mesna and or force diuresis could be use by using diuretics for high dose, minimum fluid 2L/ day.

Monitor the patient

If Yes, observe for the possible ADRs

If No

Cardiovascular toxicity, CBC, immunology, neurology: psychosis, extrapyramidal symptoms, amenorrhea, sterility, pulmonary toxicity (Eg; interstitial pneumonitis, pulmonary fibrosis), alopecia, nausea, vomiting, infectious disease, hematuria

Continue from section 72

**64.** Does the patient prescribed with Ifosfamide:

**65.** Does the patient achieve these requirements:

- **Age (>60 years):** Refer normal adult dose
- **BSA, BMI:** Calculate the dose through body weight and BSA
- **Types and stage of cancer:** Advanced (platinum-resistant), Germ cell tumor of ovary

If not, recommend

## Drug and dose based

- **Route and dose**: 1,000 to 1,200 mg/m^2^/day for 5 days (with Mesna) every 28 days for up to 6 cycles, 1.2 g/m^2^/day 30 min for consecutive 5 days, Maximum: 16 g/m^2^.
- **Drug preparation:** By using water for injections, or sterile bacteriostatic water which contains parabens and benzyl alcohol preservative shake well till the powder get dissolve (1g powder in 20ml of WFI and 3g in 60ml) to obtain the final concentrations of 50mg/ml. The solution can be further diluted to obtain the concentrations of 0.6 to 20mg/mL. Store the solutions in the large volume parenteral PAB™ bag, glass containers, via flex bags and solution will be immediately diluted to the 0.9% NS or 5% dextrose or ringer solutions.

**66.** Does the drug achieve these requirements:

Monitor the patients

**66.** Does the drug achieve these requirements:

If not, recommend

## Drug and dose based

- **Drug selection: Regimen** VIP, VeIP, TIP
- **Drug administration/ use**: As the parenteral product should be free from the foreign particles, precipitates and discolorations before use. Maintain the adequate hydrations 24 hours before and after the drug administrations.
- **Slow IV** infusion of dose over a 30 min1,000 to 1,200 mg/m^2^/day for 5 consecutive days, and repeat every 3 to 4 weeks, up to 6 cycles.

Hypersensitivity to Ifosfamide, urinary outflow obstruction, end stage of renal disease

Avoid use

**67.** Check if this formulation is contraindicated to patient:

**68.** Does the Ifosfamide concomitantly prescribed with:

Olaparib

Netupitant

CYP3A4 (Inducers or inhibitors)

COVID-19 vaccine

G-CSF

May enhance the myelosuppressive effect of Olaparib

Monitor the therapy

Administer 24 hour before or after the chemotherapy

Immunosuppressant may reduce the effectiveness of the G-CSF

May increase the concentrations of the toxic metabolites of Ifosfamide

Chemotherapy may diminish the therapeutic effect of COVID-19 vaccine

May alter the serum concentration of Ifosfamide

## 69. Abnormal clinical changes in patient:

- **Gastrointestinal:** Nausea and vomiting
- **Hematologic:** Fatigue, anemic, pale
- **Renal:** Renal toxicity**,** Hemorrhagic cystitis, hematuria without Mesna more evident
- **Hepatic:** Mild to moderate no need to adjust dose, severe impairments avoid use due to risk reduce in efficacy of drug.
- **Neurologic:** Encephalopathy, neurotoxicity
- **Respiratory:** Dyspnea, breathing problems
- **Dermatologic:** Skin rashes /angioedema/SJS/ alopecia
- **Infections:** Infectious disease

Monitor the patient

Monitor the patient

- **CBC:** Anemia, leukopenia, neutropenia
- **RFT:** S. Creatinine and urea level monitor dose adjustment or terminate the drug. Monitor S. creatinine when fluconazole used together.
- **Electrolytes:** Hyponatremia
- **LFT:** Hepatic insufficiency, increased serum bilirubin, increased serum transaminases
- **Imagination: CT scan, USG, X-ray** (tumor size reduction efficacy of drug/ other abnormalities)

## 70. Lab Investigations:

## 71. Patients care process Changes:

- **Sudden withdrawn of medications:** Encephalopathy avoid use, WBC <2,000/mm^3^, platelets <50,000/mm^3^,
- **Hepatic impairments:** Administer 25% of dose if the bilirubin >3 mg/dL
- **Renal Impairments:** Administer 75% of the normal dose if CrCl <10 mL/minute,
- **ER visit/ Readmission to ICU/ Extend hospital stay**
- **Use of blood products**
- **Dose reductions:** CrCl <10 mL/minute: administer 75% of dose

Monitor the patient

If Yes, observe possible ADRs

If No

Peripheral edema, fatigue, dizziness, hypokalemia, hypophosphatemia, diarrhea, nausea, vomiting, decreased appetite, constipation, mucositis, abdominal pain, dysgeusia, stomatitis, dyspepsia, bone marrow depression

Continue from section 80

**72.** Does the patient prescribed with Melphalan:

**73.** Does the patient achieve these requirements:

- **Age:** For the geriatric population use with the lower range of adult dose.
- **Weight, BSA, BMI:** Calculate the dose based on the body weight and BSA
- **Types and stage of cancer:** Malignant epithelial tumor of ovary**,** nonresectable epithelial carcinoma of the ovary

If not, recommend

**74.** Does the drug achieve these requirements:

If not, recommend

## Drug and dose based

- **Drug selection:** Advance stage ovarian cancer, platinum resistance
- **Dose**: Oral 0.2 mg/kg/day for 5 days, repeat this course of therapy every 4 to 5 weeks, depending on the patient level of hematologic toxicity

## Drug preparation: The drug can be reconstituted by injecting the diluent in to the vials of lyophilized powder through the sterile 20-gauge needle or large diameter needle. Vigorously agitate and obtained the clear solutions. This will result that 5mg/mL, which is further diluted with 0.9% NS to obtain 0.45 mg/mL

## Drug dispense: Low, high dose, wrong dosage form, wrong drug

- **Drug administration/ use**: **IV**: Complete infusion should be administered within 60 min, **Oral:** Administer on an empty stomach, do not mix or combine the formulations

Hypersensitivity and/ or prior resistance to Melphalan

Avoid use

**75.** Check if this formulation is contraindicated to patient:

Cisplatin

Olaparib

Nalidixic acid

Clozapine

COVID-19 vaccine

G-CSF

May enhance the myelosuppressive effect of Olaparib

May diminish Melphalan clearance

Monitor the therapy

Monitor renal function

Myelosuppressive agent may reduce the therapeutic effectiveness of these drugs

May result in an increased risk of hemorrhagic necrotic enterocolitis

May increase risk for neutropenia

**76.** Does the Melphalan concomitantly prescribed with:

## 77. Abnormal clinical changes in patient:

- **Gastrointestinal:** Nausea and vomiting, diarrhea, constipation, stomatitis, mucositis, abdominal pain, dyspepsia, alter in taste
- **Hemorrhagic:** Frequent infection, fatigue, pale skin, bleeding
- **Renal:** Acute urinary retention, pain and burning micturition
- **Hepatic impairments:** No dose adjustment is required, hepatic sinusoidal obstruction syndrome, hepatitis, jaundice

Monitor the patient

## 77. Abnormal clinical changes in patient:

- **Respiratory failure:** Dyspnea, breathing problems
- **Dermatologic:** Skin rashes /angioedema/SJS/ alopecia
- **Others:** Infectious disease, peripheral edema, fever, chill

Monitor the patient

## 78. Lab Investigations:

Monitor the patient

- - **CBC:** Anemia, leukopenia, neutropenia, thrombocytopenia
  - **RFT:** Increased blood urea nitrogen,
  - **Electrolytes:** Hypokalemia and hypophosphatemia
  - **LFT:** Increased serum transaminases
  - **Imagination: CT scan, USG, X-ray** (tumor size reduction efficacy of drug/ other abnormalities)

## 79. Patients care process Changes:

:

## Sudden withdraw/ hold of medications: If WBC <3000/mm^3^ and Platelets <100,000/mm^3^: Withhold treatment until recovery, Avoid the use of Lenograstim 24 hours before and after the myelosuppressive chemotherapeutic agents

- - **ER visit/ Readmission to ICU/ Extend hospital stay**
  - **Use of blood products**
  - **Monitor:** CBC with differential; at least one time prior to each dose, LFT, including severe infections, bleeding, and symptomatic anemia

Monitor the patient

If Yes, observe for the possible ADRs

If No

Peripheral sensory neuropathy, nausea, vomiting, anemia, fatigue, dizziness, bone marrow depression, mood swing, nephrotoxicity, pancytopenia, alopecia

Continue from section 86

**80.** Does the patient prescribed with Cisplatin:

**81**. Does the drug achieve these requirements:

## Age (≥65 years age): More usceptible to nephrotoxicity and peripheral neuropathy monitor patients and use drug with precautions

- **BSA, BMI:** BSA based
- **Types and stage of cancer:** Ovary cancer **A**dvance, Metastatic, Germ cell tumors

If not, recommend

**82**. Does drug achieve these requirements:

If not recommend

## Drug and dose based

- **Drug selection:** First line therapy for ovarian cancer
- **Route and dose**: IV: 60 to 120 mg/m^2^, BEP regimen
- **Drug preparation:** Do not use the aluminum containing containers, needles for the preparation, storage, and administration of Cisplatin sterile conditions should be maintained while preparations of drug. The drug product is diluted in 2 liters of the 5% Dextrose in half or one third parts of normal saline constitute of 37.5g of mannitol.
- Do not dilute cisplatin in just 5% Dextrose Injection, **Intraperitoneal:** Solution was prepared in warmed saline.
- **Drug administration/ use**: Infused over 6-8 hours period. IV: 75 to 100 mg/m^2^ once
- every 21 to 28 days, 75 mg/m^2^ every 21 days (in combination with Paclitaxel), Cisplatin has been infused over 30 minutes to 4 hours, at a rate of 1 mg/minute, or as a continuous infusion. Intraperitoneal: 100 mg/m^2^ on day two of 21 days programme cycle (combination with the IV and IP Paclitaxel), six cycles. Infused as the continuous infusion for the 30minutes to 4 hours’ time period and do not use rapid administrations, and intraperitoneal catheter can use for the rapid administration of intraperitoneal solution, aluminum containing. BEP regimen: 20 mg/m^2^ on days 1 to 5 every 21 days in combination for 3 cycles whereas 20mg/m^2^ in EP and 25mg /m^2^ in TPS regimen.

**Platinum based chemotherapy: Cisplatin, Carboplatin, Oxaliplatin**

May enhance the myelosuppressive effect of Olaparib

Olaparib

Paclitaxel

Doxorubicin conventional

COVID-19 vaccine

G-CSF

Vinorelbine

Docetaxel

Melphalan

Loop Diuretics

May increase the plasma concentrations of paclitaxel and enhance the myelosuppression

Monitor the therapy

Administer Cisplatin after Paclitaxel

May induce the risk of leukemia

Weigh risk and benefit carefully

Immunosuppressant may reduce the effectiveness of these drugs

Administer Cisplatin after Paclitaxel

Administer 24 hour before or after the chemotherapy

May result in an increased risk of granulocytopenia

May result in an increased risk of neuropathy

May result in a decrease in Melphalan clearance

May enhance the risk of nephrotoxic and ototoxic effect of Cisplatin

**83.** If the patient is concomitantly using these drugs with Cisplatin:

Monitor therapy

## 85. Abnormal clinical changes in patient:

- **Antiemetic:** Use highly effective antiemetic agents
- **ER vist/ ICU admission:**
- **Use of renal protectant (diuretics, hydration):** Proper hydrations administer appropriate pretreatment hydration and maintain adequate hydration and urinary output for 24 hours following cisplatin administration.
- **Use of any blood product**

Monitor the patient

Hypersensitivity to the compound and the formulation

Avoid use

**84. Does the patient have:**

If Yes, observe the possible ADRs

If No

Peripheral sensory neuropathy, nausea and vomiting, anemia, fatigue, dizziness, bone marrow depression, mood swing, nephrotoxicity, pancytopenia, constipation, diarrhea, bleeding complication, hemorrhage, Increase in ALP and AST

Continue from section 94

**86.** Does the patient prescribed with Carboplatin:

**87**. Does the patient achieve these requirements: :

- **Age** **(>60 years):** More susceptible to nephrotoxicity and peripheral neuropathy monitor patient and use drug with precautions
- **BSA, AUC, GFR:** Calculate the dose based on the BSA or Calvert formula (Dose (mg) = AUC x (GFR + 25)
- **Types and stage of cancer:** Ovarian cancer, Advanced (palliative treatment of recurrent disease, including patients previously treated with Cisplatin)

If not, recommend

**88**. Does the drug achieve these requirements:

If not, recommend

## Drug and dose based

- **Drug selection:** First line chemo therapy in platinum sensitive ovarian cancer

## Drug preparation: Do not use the Aluminium containing containers, needles for the preparation, storage, and administration of Carboplatin sterile conditions should be maintained while preparations of drug. Prepare the drug with adopting the safety technique; Double gloves, using separate cabin, protect the eyes and respiratory tract, transfer the reconstitute solution through IV line from the primed.

## Carboplatin injection 10 mg/mL will be supplied as ready to use in 5, 15, 45 and 60ml vials, which further diluted up to 0.5 mg/mL with 5% Dextrose in Water (D5W) or 0.9% Sodium chloride injection.

- **Drug administration/ use: IV** 30min to 60 min after Paclitaxel, **Early-stage epithelial, adjuvant therapy:** (AUC 7.5 IV infusion over 30 minutes in combination with Paclitaxel 175 mg/m^2^ IV infusion over 3 hours every 21 days for 3 or 6 cycles (off-label dosage), AUC 6 IV infusion over 60 minutes after Paclitaxel 175 mg/m^2^ IV infusion over 3 hours every 3 weeks for 4 cycles (off-label dosage), infuse over 15 to 60 minutes sometimes protocol of 24 hours administration

May enhance the myelosuppressive effect of Olaparib

Olaparib

Taxane

Aminoglycosides

G-CSF

COVID-19 vaccine

Warfarin

May increase the plasma concentrations of taxel and enhance the myelosuppression

Monitor the therapy

Administer Carboplatin after Taxane

May enhance nephrotoxicity of aminoglycosides and aminoglycoside may enhance ototoxicity of Carboplatin

Monitor therapy especially high dose of Carboplatin

Immunosuppressant may reduce the effectiveness of these drugs

Administer 24 hour before or after the chemotherapy

May increase the risk of bleeding and enhance INR level

**89.** Does the Carboplatin concomitantly prescribed with:

Monitor the therapy

**91.** Abnormal clinical changes in patient:

- **Gastrointestinal:** Nausea and vomiting, taste alteration
- **Cardiovascular:** Tachycardia, atrial fibrillations, palpations
- **Neurological:** Peripheral neuropathy,
- **Hematologic:** Myelosuppression,
- **Renal:** Acute urinary retention**,** hemolytic uremic syndrome
- **Hepatic impairments:** Increase in ALP level
- **Respiratory failure:** Dyspnea, pulmonary embolism, cough, pleural effusion
- **Dermatologic:** Skin rashes /angioedema/SJS/ alopecia
- **Infections:** Pneumonia, UTI, Sepsis, septic shock, ototoxicity, tinnitus

Monitor the patient

Severe allergic reactions to Cisplatin, platinum-containing compounds, or Mannitol (certain preparations contain Mannitol), severe bone marrow depression, significant bleeding.

Avoid use

**90.** Check if this formulation is contraindicated to patient:

## 92. Lab Investigations:

- **CBC:** Anemia, leukopenia, neutropenia
- **RFT:** GFR, Creatinine clearance**,** increased blood urea nitrogen, serum creatinine
- **Endocrine metabolic:** electrolytes imbalance; hypocalcemia, hyponatremia, hypomagnesemia, hypokalemia
- **LFT:** Alkaline phosphate, aspartate aminotransferase
- **Imagination: CT scan, USG, X-ray** (tumor size reduction efficacy of drug/ other abnormalities)

Monitor the patient

**93**. Patients care process Changes:

- **Sudden withdraw/ hold of medications:** Platelets <50,000 cells/mm^3^ or ANC <500 cells/mm^3^: Administer 75% of the usual dose.
- **Renal Impairment:** Baseline CrCl 41 to 59 mL/minute: Initiate at 250 mg/m^2^ and adjust subsequent doses based on bone marrow toxicity, CrCl 16 to 40 mL/minute: Initiate at 200 mg/m^2^, CrCl ≤15 mL/minute: There are no dosage adjustments provided in the manufacturer's labeling. GFR >50 mL/minute: No dosage adjustment is necessary. GFR 10 to 50 ml/minute: Administer 50% of the usual dose, GFR <10 mL/minute: Administer 25% of the usual dose, hemodialysis: Administer 50% of the usual dose.
- **ER visit/ Readmission to ICU/ Extend hospital stay**
- **Use of blood products**
- **Dose reductions:** Renal impairment patients, mylosuppressions, neuropathy
- **Monitor:** At least monthly monitor the peripheral blood counts prior to the therapy, monitor for the neutropenia due to the Clozapin**e**, monitor for the therapeutic effects of vaccine, neurologic examination, Monitor the renal parameter
- **Drug to be avoid use together:** After developing grade 3 to 4 neuropathy discontinue or avoid the use of Lenograstim 24 hour before and after the cytotoxic agents.

Monitor the patient

If Yes, observe possible ADRs

If No

Peripheral sensory neuropathy, nausea and vomiting, anemia, fatigue, dizziness, bone marrow depression, mood swing, thrombocytopenia, nephrotoxicity, bleeding complication, Increase in ALP and AST, paresthesia, backache, cough, dyspnea, constipation, diarrhea, fever

Continue from section 102

**94.** Does the patient prescribed with Oxaliplatin:

**95**. Does the patient achieve these requirements:

- **Age (>60 years):** Refer normal adult dose
- **BSA, BMI:** Dose calculation according to the BSA, and consider when renal and hematological impairment occurs.
- **Types and stage of cancer:** Advance stage EOC ovarian cancer, with metastasis to the peritoneum or colon

If not, recommend

**96**. Is the drug achieve these requirements:

If not, recommend

## Drug and dose based

- **Drug selection:** Advanced ovarian cancer:
- **Route and dose**: **IV**
- Oxaliplatin (130 mg/m^2^ intravenous, day 1) and capecitabine (850 mg/m^2^ orally twice daily, days 1–14), repeat every 3 weeks.
- Oxaliplatin 85mg/m^2^ and topotecan 2-4mg/m^2^ on day 1, 8 and 15 of 28 days, day 1 Oxaliplatin and followed by the Topotecan on day 8 and 15 of 28 days cycle.

Oxaliplatin 75mg/m^2^ on day 1, IV and 50-75mg/m^2^ on day 2, IP 3–4-week cycle.

- **Drug administration/ use:**

Infusion line most be flushed with D5W prior to infusion of any concomitant drug and infused with D5W 250 to 500ml over 2 hours and up to 6 hours after dilution can be used, concentration must be 0.2 to 0.7 mg/ml.

The lyophilized powder is reconstituted by adding 10 mL (for the 50 mg vial) or 20 mL (for the 100 mg vial) of Water for Injection, USP or 5% Dextrose Injection, USP. Do not administer the reconstituted solution without further dilution.

Hypersensitive to the Platinum, neuropathy grade 3 and grade 4

Avoid use

**97.** Does the patient has:

- **Gastrointestinal:** Nausea and vomiting, diarrhea, constipation, anorexia, organomegaly
- **Cardiovascular:** Peripheral edema, chest pain
- **Neurological:** Peripheral neuropathy, paresthesia
- **Hematologic:** Lethargy, fatigue, risk of infection, bleeding risk
- **Renal:** Acute urinary retention**,** dysuria
- **Hepatic:** increase in ALP level and no dose adjustment is required
- **Respiratory failure:** Cough, dyspnea
- **Dermatologic:** Skin rashes, alopecia

Avoid use

**98.** Abnormal clinical changes in the patients

May enhance the myelosuppressive effect of Olaparib

Olaparib

Taxane/ Topotecan

QT prolonging

G-CSF

Anticoagulant

Oxaliplatin may increase the plasma concentrations of these drugs and enhance the myelosuppression

Monitor the therapy

Administer platin after these agents and monitor for the risk

May increase the risk of QT by Oxaliplatin

Monitor therapy especially high dose of Oxaliplatin

Immunosuppressant may reduce the effectiveness of G-CSF

Administer 24 hour before or after the chemotherapy

May increase the risk of bleeding and enhance INR level

**99.** Does the Oxaliplatin concomitantly prescribed with:

Monitor the therapy

## 100. Lab Investigations:

- **CBC:** thrombocytopenia, anemia, neutropenia
- **RFT: GRF, Creatinine clearance,** Increased serum creatinine
- **LFT:** Alkaline phosphate and AST elevation
- **Imagination: CT scan, USG, X-ray** (tumor size reduction efficacy of drug/ other abnormalities)

Monitor the patient

**101**. Patients care process Changes

- **Sudden withdraw/ hold of medications:** Severe neuropathy
- **Renal:** If CrCl < 30 mL/min reduce the Oxaliplatin dose to 65 mg/m^2^_,_ reduce dose by 30% in hemodialysis patients, and administer after hemodialysis**.**
- **Use of blood products/ or emergency visit/ or Readmission to ICU/ Extend hospital stay**
- **Dose reductions:** Renal impairment
- **Monitor:** At least monthly monitor the peripheral blood counts prior to the therapy, neurologic examination, monitor the renal parameter
- **Drug to be avoid use together:** After developing grade 3 to 4 neuropathy discontinue avoid the use of Lenograstim 24 hour before and after the cytotoxic agents

Monitor the patient

If Yes, Observe for the possible ADRs

If No

Peripheral edema, alopecia, rashes, bullous eruption, GI issue, hematological issues, infectious disease, neuropathy, serum creatinine raised, hepatotoxicity, respiratory issue

Continue from section 110

**102.** Does the patient prescribed with Gemcitabine:

**Antimetabolites:**

- **Pyrimidine analogue:** **Gemcitabine & Capecitabine**

**103.** Does the patient achieve these requirements:

- **Age (>60 years):** Refer normal adult dose
- **Weight, BSA, BMI:** Dose calculation according to the BSA and body weight, and consider when hepatic and renal impairment occurs.
- **Types and stage of cancer:** Advance stage ovarian cancer

If not, recommend

**104**. Does the drug achieve these requirements:

If not recommend

## Drug and dose based

**Gemcitabine**

- **Drug selection:** In combinations with carboplatin replace after the six months completions of the Platinum based therapy.
- **Route and dose of**: **IV:** 1,000 mg/m^2^ either single or in combination with Carboplatin
- **Drug preparation**: The lyophilized powder vials can be reconstituted with diluent (0.9% NS) to obtain the concentrations of 40mg/ml. The dose of 200mg of vial will be reconstituted with 5ml of diluent, whereas 1-gram vials with 25ml of diluent then resulted concentration of gemcitabine will be 38mg/ml, which needs the displacement volume calculations to adjust the concentrations 0.26 ml diluent will be added to the 200mg vial and 1.3ml in 1gm vial to obtain the 5.26ml and 26.3ml volume in each.
- It is necessary complete removal of the drug 200mg and 1gm from each vial. This reconstituted solution will be further diluted with 0.9% NS to obtain concentrations of 0.1mg/ml which is ready for infusion.
- Diluents to be used for predations of solutions 50 to 500ml of 0.9% NS, D5W
- **Drug administration: IV:**
- IV: 1,000 mg/m^2^ over 30 minutes days 1 and 8; repeat cycle every 21 days (in combination with Carboplatin) or single: 1,000 mg/m^2^ over 30 to 60 minutes days 1 and 8; repeat cycle every 21 days
- Infusion time should not be longer than the 30 minutes, longer the infusion time more the chance of getting toxicity.

**Capecitabine route and dose:** Oral:1000mg/m^2^ BD from day 1 to day 14, 21 days cycle. (Till unacceptable toxicity or disease progression)

Hypersensitivity or idiosyncratic reactions to gemcitabine or Capecitabine any component of the product

Avoid use

**105.** Does the patient has:

May enhance the myelosuppressive effect of Olaparib

Olaparib

Bleomycin

Clozapine

G-CSF

COVID-19 vaccine

Warfarin

May enhance the risk of pulmonary toxicity of Bleomycin

Monitor the therapy

May increase the risk of Neutropenia due to Clozapine

Immunosuppressant may reduce the effectiveness of these drugs

Administer 24 hour before or after the chemotherapy

May enhance the anticoagulant effect of Warfarin

**106.** Does the Gemcitabine concomitantly prescribed with:

Monitor the therapy

## 108. Lab Investigations:

- **CBC:** Neutropenia, leukopenia, anemia, thrombocytopenia
- **RFT:** Proteinuria, increased blood urea nitrogen
- **Electrolytes:** Electrolytes imbalance
- **LFT:** AST, ALT, ALP, bilirubin
- **Metabolic/ Endocrine changes**: Hypokalemia, hyponatremia, hypomagnesaemia, hyperglycemia
- **Imagination: CT scan, USG, X-ray** (tumor size reduction efficacy of drug/ other abnormalities**)**

Monitor the patient

**109**. Patients care process Changes:

- **Sudden withdraw/ hold of medications**: If severe hepatotoxicity, severe ANC and thrombocytopenia
- **Renal Impairment:** No dosage adjustment necessary in renal impairment and after 6-12 hours Gemcitabine infusion followed by hemodialysis.
- **Hepatic Impairment**: Serum bilirubin >1.6 mg/dL: Use initial dose of 800 mg/m^2^ can increase the dose if patients tolerated, Transaminases elevated (with normal bilirubin): No dosage adjustment
- **Hematologic:** Day 1: ANC ≥1,500/mm^3^ and platelet count ≥100,000/mm^3^: Administer 100% of full dose, ANC <1,500/mm^3^ or platelet count <100,000/mm^3^: Delay treatment cycle. Day 8: ANC ≥1,500/mm^3^ and platelet count ≥100,000/mm^3^: Administer 100% of full dose, ANC 1,000 to 1,499/mm^3^ or platelet count 75,000 to 99,999/mm^3^: Administer 50% of full dose, ANC <1,000/mm^3^ or platelet count <75,000/mm^3^ the hold dose
- **ER visit/ Readmission to ICU/ Extend hospital stay**
- **Use of blood products**
- **Dose reductions:** Renal impairment conditions
- **Monitor:** Pulmonary function tests, renal function, liver function

Monitor the patient

If Yes, observe for the possible ADRs

If No

Hot flashes, ocular effects, thromboembolic events, uterine malignancies, flushing, hypertension, peripheral edema, vasodilation, DPT, chest pain

Continue from section 118

**110.** Does the patient prescribed with Tamoxifen:

**Antiestrogen: Tamoxifen**

**111**. Does the patient achieve these requirements: uirements

- - **Age (>60 years)**: Refer normal adult dose
  - **Weight, BSA, BMI:** Dose calculation according to the BSA and body weight. Renal impairments conditions consider dose adjustment
  - **Types and stage of cancer**: Advanced and/or recurrent, malignant epithelial tumor of ovary

If not, recommend

- **Gastrointestinal:** Nausea and vomiting**,** stomatitis, diarrhea
- **Neurological:** Drowsiness, paresthesia
- **Hematologic:** Weakness, anemic, pale
- **Renal:** Acute renal failure, dysuria, oliguria, maturation urgency
- **Hepatic:** Transaminases elevated, bilirubin
- **Ophthalmic:** Discoloration of tears, eye infections
- **Respiratory:** Dyspnea, flu-like symptoms
- **Dermatologic:** Hyperpigmentation, atrophic striae, erythema, exfoliation of the skin on the palmar and plantar surfaces of the hands and feet, hyperkeratosis, localized vesiculation, skin rash, skin sclerosis, alopecia, nailbed changes
- **Others:** Injection site reaction, hematuria, fever, infection peripheral edema

Monitor the patient

## 107. Abnormal clinical changes in patient:

May increase the serum concentration of CYP3A4 substrates

Aprepitant:

Vitamin K Antagonists

QT-prolonging Agents

Hydroxychloroquine

May increase the plasma concentrations of vitamin K antagonist

Monitor the therapy

Avoid use together

Additional risk of QT prolongation may increase

Monitor therapy

May enhance the renal toxicity of Hydroxychloroquine

**114.** Does the Tamoxifen concomitantly prescribed with:

**115.** Abnormal clinical changes in patient:

- **Gastrointestinal:** Diarrhea, nausea, vomiting, abdominal pain, constipation, dyspepsia
- **Cardiovascular:** Flushing, hypertension, peripheral edema, vasodilation, DVT, chest pain
- **Neurological:** Depression, fatigue, mood changes, pain
- **Hematologic:** Weakness, anemia, risk of bleeding
- **Renal:** Oliguria, urinary incontinence
- **Hepatic:** Hepatitis, hepatic failure, jaundice, hepatomegaly
- **Ophthalmic:** Blurred vision, conjunctivitis, eye pain, transient blindness
- **Respiratory:** Dyspnea, bronchitis, sinusitis
- **Dermatologic:** Skin changes, skin rash
- **Metabolic/:** Amenorrhea, fluid retention, hot flash, weight loss, decreased appetite, DM
- **Others:** Irregular menses, vaginal discharges, hemorrhage, infection, sepsis
  - **Renal Impairments:** Renal impairment can be worsened
  - **Hepatic impairments:** Hepatitis, hepatic failure
  - **Ophthalmic:** Blurred vision, conjunctivitis, eye pain, transient blindness
  - **Respiratory failure:** Dyspnea, Bronchitis, sinusitis
  - **Dermatologic:** skin changes, skin rash
- **Infections:** Infection, sepsis
- **Metabolic/ Endocriane changes:** Amenorrhea, fluid retention, hot flash, weight loss, Decreased appetite, DM
- **Others:** Irregular menses, vaginal discharges, hemorrhage,

Monitor the patient

- - **RFT:** Serum creatinine, BUN
  - **Electrolytes:** Electrolytes imbalance
  - **LFT:** Increased serum aspartate aminotransferase (5%), increased serum bilirubin
  - **Metabolic/ Endocrine changes**: Hypertriglyceridemia, Hypercalcemia, Hyperlipidemia, Hypercholesterolemia, Hyperglycemia
  - **Imagination: CT scan, USG, X-ray** (tumor size reduction efficacy of drug/ other abnormalities)

Monitor the patient

## 116. Lab Investigations:

**117**. Patients care process changes:

- **Sudden withdraw/ hold of medications**:
- **Hematologic:** Use alternative to the Doxycycline if toxicities persist, vitamin B6
- **ER visit**/ **Readmission to ICU/ Extend hospital stay**
- **Use of blood products**
- **Monitor:** CBC, LFT, pregnancy test before starting of therapy, ophthalmic examination, long term therapy so monitor adherence
- **Drug to be avoided use together:** Strong and moderate inhibitor and inducer of CYP3A4

Monitor the patient

112. Does the drug achieve these requirements:

If not, recommend

## Drug and dose based

- **Drug selection:** Recurrent, malignant epithelial tumor of ovary
- **Route and dose**: **Oral** 20 mg Once daily, Range 20-120mg
- **Drug storage:** Store tamoxifen at room temperature and protect from light
- **Drug dispense:** low, high dose, wrong dosage form, wrong drug
- **Drug administration/ use:** Oral: Administer tablets 20mg per oral once in a day, if dose greater than 20mg per day then divide the dose into twice a day. Do not chew or crush the tablet.

Known hypersensitivity to tamoxifen or concurrent warfarin therapy or history Tamoxifen induced DVT or pulmonary embolism

Avoid use

**113.** Does the patient has:

**119**. Does the patient achieve these requirements:

- **Age (> 60 years)**: Normal adult dose
- **Weight, BSA, BMI:** Dose calculation according to the BSA and body weight, and consider if hepatic and renal impairment.
- **Types and stage of cancer:** Advanced, germline or somatic BRCA -mutated, recurrent, maintenance therapy, not previously treated with PARP inhibitors
- **Not recommended:** Repeating PARPi therapy in EOC, and newly diagnosed stage I -II (early stage) EOC

If not, recommend

**120**. Does the drug achieve these requirements:

If not, recommend

## Drug and dose based

- **Drug selection: Patients diagnosed with epithelial ovarian, tubal, or primary peritoneal cancer (EOC) who have not previously received a poly (ADP-ribose) polymerase inhibitor (PARP),** somatic BRCA -mutated **first-line maintenance therapy (monotherapy)**

**Route and dose: Oral**

- **Recently detected stage III-IV EOC with partial or complete response to 1^st^ line platinum-based chemotherapy: Treated with PARPi maintenance therapy**
- **Tab Olaparib** 300 mg orally every 12 hours for 2 years: (for those with germline or somatic pathogenic or likely pathogenic variants in BRCA1 or BRCA2 genes)
- **Tab Niraparib** 200-300 mg orally daily for 3 years: (all women) in high-grade serous (HGS) or endometrioid ovarian cancer.
- **Tab Rucaparib:** Post recurrence condition start dose 600 mg BD, (two 300mg tab) continue until tolerance, 1^st^ dose reduction 500mg BD (two 250mg tab), 2^nd^ dose 400mg BD, (two 200mg tab), 3^rd^ dose reduction 300mg OD (one 300mg tab)
- **Olaparib and Bevacizumab** **combination:** could be recommend to the patients with stage III-IV HGS or endometrioid ovarian cancer and germline or somatic pathogenic or BRCA positive, unstable gene
- **Ovarian cancer, recurrent, maintenance therapy:** **Oral:** PARPi as the second line more regardless of BRCA positive, continue until unacceptable toxicity, dose reduction, disease progression
- **Tablet Olaparib**: 300 mg twice daily**, Rucaparib** 600 mg/BD, **Niraparib** 200-300 mg OD.

**Note:** Missed dose: If dose is missed then skip the dose and go for the next regular dose not double the dose.

Infusion time should not be longer than the 30 minutes. Longer the infusion time more the chance of getting toxicity.

Hypersensitivity to PARPi and its formulation ingredients

Avoid use

**121.** Does the patient has:

May increase myelosuppressive activity of PARPi

Myelosuppressive

CYP3A4 Inducers/ Inhibitors

CoVID-19 vaccine

May alter the serum concentration of PARPi

Monitor the therapy

Avoid use together or adjust dose

**122.** If the patient is concomitantly using these drugs with PARPi:

May reduce the effectiveness of Covid 19 vaccine

Monitor the therapy

If Yes, Observe for the possible ADRs

If No

Hypertension, peripheral edema, venous thromboembolism, hypertensive crisis/hypertensive encephalopathy, anorexia, diarrhea, dysgeusia, nausea, stomatitis,

Continue from section 126

**118.** Does the patient prescribed with PARPI:

**:**

**PARP Inhibitor: Olaparib, Niraparib, Rucaparib**

## VEGF inhibitors: Bevacizumab

If Yes, Observe for the possible ADRs

If No

Hypertension**,** peripheral edema, venous thromboembolism, hypertensive crisis and or hypertensive encephalopathy, decreased appetite, diarrhea, dysgeusia, nausea, stomatitis, ovarian failure

Continue from section 134

**126.** Does the patient prescribed with Bevacizumab:

## 124. Lab Investigations:

- **CBC:** Blood counts decrease
- **RFT:** Increased serum creatinine, proteinuria, increased blood urea nitrogen
- **Electrolytes:** Electrolytes imbalance
- **LFT:**  Hyperbilirubinemia, abnormal LFT, transaminases elevated, bilirubin
- **Metabolic/ Endocrine changes:**  Reductions in electrolytes, cachexia, Increase in blood glucose
- **Imagination: CT scan, USG, X-ray** (tumor size reduction efficacy of drug/ other abnormalities**)**

Monitor the patient

**125**. Patients care process changes:

- **Sudden withdraw/ hold of medications**: If pneumonitis, Neutropenia grade 4 and fever for 5-7 days, Hb< 8g/dl or platelets< 1lakh cells/mm^3^_,_ then hold drug for 28 days and followed by dose reduction, use GCSF stimulator. Risk of bleeding or consistent thrombocytopenia
- **Hepatic Impairment:** If the bilirubin and AST level increased by 1.5 to 3 times of upper limits of normal value, initiate with reduce dose.
- **Renal Impairment:** If CrCl 31- 50 ml/min then reduce the dose, and if the CrCl less than 30 then avoid the drug.
- **ER visit/ Readmission to ICU/Extend hospital stay**
- **Use of blood products:** Hb < 8g/dl transfusion of RBC may require until the symptoms get relief. If platelets count <10 thousand then go for the platelet transfusion and withheld concomitant use of blood thinning agents.
- **Dose reductions:** Anemia if Hb<8g/dl, Thrombocytopenia, hepatotoxicity, weight loss >5%.
- **Monitor:** BRCA-mutation status for maintenance therapy of advance ovarian cancer as first line monotherapy, proteinuria/nephrotic syndrome, CBC, PT, INR
- **Drug to be avoid use together:** Avoid CYP3A4 inducer and inhibitors
- **Drug use process:** take with or without meal, do not chew or crush tablet, and don’t take the tablet having leakage or deformed, do not replace the tablet with capsule dosage form and vice-versa

Monitor the patient

**123.** Abnormal clinical changes in patient:

- **Gastrointestinal:** Abdominal pain, diarrhea, dysgeusia, nausea, stomatitis constipation, taste altered
- **Cardiovascular:** Heart failure, tachycardia, chest pain, MI
- **Neurological:**  Anxiety, dizziness, fatigue, headache
- **Hematologic:** Weakness, pallor, breathlessness
- **Renal:** Renal fistula, renal thrombotic microangiopathy
- **Hepatic:** Portal vein thrombosis, liver failure, jaundice, cholecystitis, cholelithiasis
- **Ophthalmic:** Vision blurred, visual impairment, cataract
- **Respiratory:** Aspiration pneumonia, pulmonary embolism, URTI
- **Dermatologic:** Pruritus, rashes, dry skin
- **Others:** Arthralgia, asthenia, back pain, myalgia, proteinuria, urinary tract infection, thyroid disorders, treatment ineffectiveness, appetite, Influenza

Monitor the patient

**128**. Does the drug achieve these requirements:

If not, recommend

## Drug and dose based

- **Drug selection:** First-line treatment with combination
- **Route and dose**: **IV** 15 mg/kg every 3 weeks (with Carboplatin & Gemcitabine combination for 6 to 10 cycles or with Carboplatin and Paclitaxel for 6 to 8 cycles) then continue with Bevacizumab (monotherapy), for a total of up to 22 cycles or until disease progression
- 1^st^ line maintenance therapy: 15 mg/kg every 21 days (with Olaparib combinations), after following the 1^st^ line if able to obtain complete or partial response, platinum-based chemotherapy. The Bevacizumab should be given for 15 months (this time counts the combination with other antineoplastic agents (chemo) as well as maintenance therapy
- **Drug preparation**: Adopt the suitable aseptic conditions for the preparation of the Bevacizumab dose. This parenteral drug products should be inspected visually and make ensure that free from the foreign particles and discolorations before administrations. From the Bevacizumab vial the required amount of drug is withdraw and dilute to the 100ml solution of 0.9% sodium chloride, discard the unused product in the vial, as the drug products do not contain any preservers.
- **Note:** Don’t mix or administer with the dextrose solutions

## Drug administration/ use: IV: Infuse the initial dose over 90 minutes and if first dose tolerated then second dose given over the time 60 min and 3^rd^ time 30min infusion if second dose tolerated, at rate of 0.5 mg/kg/minute

- **Note**: Administer as the IV Infusions only, should not be used given as IV push or bolus. Don’t start the Bevacizumab minimum of 28 days of surgery incisions has totally healed.

May increase cardiotoxic effect of Anthracyclines

Anthracyclines

Clozapine

Olaparib

Sorafenib

Sunitinib

May enhance the risk of neutropenia of Clozapine

Avoid combination

Monitor therapy

**129.** Does the Bevacizumab concomitantly prescribed with:

Myelosuppressive agent may enhance the myelosuppressive effect of Olaparib

Monitor therapy

Bevacizumab may enhance the foot and skin reaction of Sorafenib

Monitor therapy

Sunitinib may enhance the hemolytic anemia of Bevacizumab, and Bevacizumab may enhance the hypertensive effect of Sunitinib

Avoid combination

**130.** Does the patient has:

Resistance or hypersensitivity to Bevacizumab, Chinese hamster ovary cell products

Avoid use

**127.** Does the patient achieve these requirements:

- **Age (>60 years):** Refer normal adult dose.
- **Weight, BSA, BMI:** Dose calculation according to the BSA and body weight, and consider when hepatic and renal impairment occurs.
- **Types and stage of cancer:** Advanced, germline or somatic BRCA -mutated, recurrent ovarian cancer.

If not, recommend

**Supportive therapy**

**133**. Patients care process changes:

- **Sudden withdrawn/ hold of medications**: Sever GI perforation, heart failure, hemoptysis, sever hypertension, wound healing reduced, venous or arterial thromboembolism
- **Renal Impairment:** Nephrotic syndrome: Discontinue Bevacizumab. Proteinuria ≥2 g/24 hours in the absence of nephrotic syndrome: Withhold bevacizumab until proteinuria <2 g/24 hours

## ER visit/ Readmission to ICU:

- **Use of blood products:** High blood loss
- **Monitor:** Proteinuria/nephrotic syndrome, infusion reaction, BP monitor, PT, INR
- **Drug to be avoid use together:** Avoid use together anthracycline
- **Drug use process:** Mild clinically insignificant then alter the infusion rate, if clinically significant holds and infuse after the symptoms resolved. If severe: Discontinue and medical managements with the corticosteroid, epinephrine, bronchodilators or oxygen therapy.

Monitor the patient

## 132. Lab Investigations:

- **CBC:** Blood counts decrease
- **RFT:** Proteinuria, increased blood urea nitrogen
- **Electrolytes:** Electrolytes imbalance
- **LFT:** Hyperbilirubinemia, abnormal LFT, elevated transaminases and bilirubin
- **Metabolic/ Endocrine changes:** Reductions in electrolytes, cachexia, increase in blood glucose
- **Imagination: CT scan, USG, X-ray** (tumor size reduction efficacy of drug/ other abnormalities**)**

Monitor the patient

**131.** Abnormal clinical changes in patient:

- **Gastrointestinal:** Abdominal pain, decreased appetite, diarrhea, dysgeusia, nausea, stomatitis, constipation, GI perforation, fistula of bile duct, gastrointestinal perforation, gingival hemorrhage, mucosal ulcer
- **Cardiovascular:** Hypertension, peripheral edema, venous thromboembolism, hemorrhage, hypotension
- **Neurological:** Anxiety, dizziness, dysarthria, fatigue, headache, insomnia, myasthenia, voice disorder
- **Hematologic:** Weakness, Anemic
- **Renal:** Renal fistula, renal thrombotic microangiopathy
- **Hepatic:** Portal vein thrombosis liver failure, jaundice, cholecystitis, cholelithiasis
- **Ophthalmic:** Disease of the lacrimal apparatus
- **Respiratory:** Cough, dyspnea, epistaxis, oropharyngeal pain, sinusitis, nasal congestion
- **Dermatologic:** Exfoliative dermatitis, xeroderma
- **Others:** Pelvic pain, proteinuria, urinary tract infection, decreased appetite, dehydration, infection

Monitor the patient

**Antiemetics:** 5HT3RA, NK1RA, DRA

5HT3 antagonist+ Neurokinin-1 receptor antagonist+ Dexamethasone+ Olanzapine

Cisplatin, Anthracycles with Cyclophosphamide)

Select the combination of

**134**. Does the patient is under highly emetogenic:

Cyclophosphamide (<1,500 mg/m2)

Daunorubicin

Doxorubicin

Ifosfamide (2g/m^2^ per dose)

Carboplatin based regimen

**135.** Does the patient is under moderately emetogenic:

5HT3 antagonist may not be recommend instead Palonosetron

Non-carboplatin-based regimen

of

Single antiemetic

Bevacizumab

Bleomycin

Daratumumab

Trastuzumab

Vinblastine

Vincristine

Vinorelbine

**136**. Does the patient under minimal or minor emetogenic:

Single dose 5HT3 antagonist can be recommended

Docetaxel

Etoposide

Gemcitabine

Methotrexate

Paclitaxel

PL Doxorubicin

**137.** Does the patient prescribed with 5HT3 antagonist:

Ondansetron

Palonosetron

Monitor the ADRs: Constipation, xerostomia, headache, serotonin syndrome, palpitation, restlessness

If yes

If No

**Ondansetron:** 8-24 mg single or divided

8mg IV or oral 8mg tablet given prior to chemo BD 1^st^ dose given prior to chemotherapy or single dose of 32mg IV, 30 min prior to dose of chemo

**Palonosetron:** 0.25- 0.75 mg IV or 0.5mg oral before 30 min of 1^st^ chemotherapy

**138**. Does the dose is in the range:

If not, Advise in recommended range

Continue the section 140

If No

**139.** Does the 5HT3 antagonist concomitantly prescribed with:

Monitor the therapy carefully

Increased risk of QT interval prolongation

Increased the risk of serotonin syndrome

CYP3A4 Inhibitors: (Fluconazole, Amiodarone, Clarithromycin, Erythromycin, Diltiazem, Itraconazole, Ketoconazole), Ciprofloxacin

Fluoxetine, Domperidone, Amisulpride

Tramadol

Metformin

Apomorphine

Monitor ECG

May increase the serum concentration of metformin.

If severe, discontinue and treat for sign and symptoms of serotonin syndrome

Avoid concomitant use of 5HT3 antagonist and Apomorphine

May enhance the hypotensive effect of Apomorphine.

**140.** Does patient prescribed with Palonosetron:

Monitor for this possible ADRs: Constipation, flatulence, headache, bradycardia, palpitation, tachycardia, dizziness,

If Yes

If No

Continue the section 142

142

If No

If yes

If No

**141.** Check if the patient is using these drugs concomitantly palonosetron:

Lithium, Granisetron, Morphine, Tramadol

**142.** Does patient is using Neurokinin-1 (NK-1) RA:

Monitor for risk of serotonin syndrome. Discontinue use of Palonosetron and begin supportive treatment if the patient exhibits signs and symptoms of serotonin syndrome: hyperreflexia, clonus, hyperthermia, diaphoresis, tremor, autonomic instability, mental status

Observe for the possible ADRs: fatigue, headache, neutropenia, hypotension, palpitations, bradycardia, constipation, diarrhea, dyspepsia

**143.** Does the dose is in the range:

**For Acute Emesis**

- Aprepitant: 125 mg the day or day of chemo, 30min to 1 hour before chemo and
- Fosaprepitant: 150 mg IV OD on the day of chemotherapy, 30 min before

**Delayed emesis**

- Aprepitant: 80mg PO OD for two days post-chemo, or non if fosaprepitant is used.
- Rolapitant: 180 mg orally once on the day of chemotherapy
- Netupitant: 300 mg Netupitant/ 0.5 mg palonosetron orally once on the day of chemotherapy

If not, advise in recommended range

Alprazolam

Olaparib

Amiodarone

Domperidone

Fluconazole

Oral contraceptive

**144.** Does CYP3A inhibiters (Aprepitant) concomitantly prescribed with:

May increase Alprazolam exposure.

Discontinue Domperidone if the patient experiences dizziness, palpitations, syncope, or seizure

Consider appropriate dose reduction of Alprazolam

May result in increased Amiodarone exposure.

May increased Olaparib exposure

Increased risk of QT prolongation.

May result in increased Aprepitant exposure and risk for toxicity.

Reduce Olaparib dosage to 150 mg twice daily

Alternative recommended Fosaprepitant 150mg have less inhibition effects

May result in increased Aprepitant exposure and risk for toxicity.

May decrease effectiveness of oral contraceptives

Nonhormonal form of birth control during treatment and for at least 1 month after the last dose

If used together with Pimozide

If hypersensitive to the Aprepitant

**145**. Does the patient contraindicated to the Aprepitant:

Avoid the drug use alone or combination form

If Yes

Monitor drowsiness, dystonic reaction, fatigue, restlessness, hypertension, diarrhea, urinary incontinence, confusion, dizziness, hallucination, headache

Continue the section 152

If No

**146.** Does the patient prescribed with Domperidone:

Continue the section 147

**150.** If the patient is using Metoclopramide for:

**151.** Does the Metoclopramide concomitantly prescribed with:

If yes, Continue and observe for the possible ADRs

If No

**147.** Does the dose is in the range:

**Domperidone:** 10-30mg/ Day, in divided doses, before meal

**Metoclopramide:** 10mg before chemotherapy or 10 mg every 6 hours SOS

**Prochlorperazine:** 5-10mg OD/ IV/PO**-** before chemo, 10mg every 6 hour for low emetogenic risk

**Olanzapine: for acute and delay emesis:** Oral 5-10mg on day of chemo, day 1 followed by 5-10mg OD day 2 to 4. For high emetic combined with other antiemetics.

If not, recommend in same range

**148**. Does the Domperidone concomitantly prescribed with:

Ketoconazole, Fluconazole

Amiodarone,

Clozapine,

Alprazolam,

GRH,

Levofloxacin,

Clarithromycin, Verapamil,

Amitriptyline,

Moxifloxacin, Ranitidine,

Antipsychotic Agents

May increase the risk of QT interval prolongation of Domperidone

Avoid combination serious cardiac effect can occur

Monitor patients for serious cardiac effects, ECG, if present discontinue the Domperidone

Reduce dose frequency to OD from BD

**149.** If the patient having:

May increase risk of QT interval prolongation

Monitor the therapy, ECG

Use is contraindicated

10 mg before chemo and 10mg every 6 hours after chemo

10 to 20 mg 4 times daily on post chemotherapy days 2 through 4; given in combination with Dexamethasone

Antipsychotics or CNS depressants

may result in an increased risk of EPS

If prescribed, consult prescriber

Recommended prescriber if not followed

May diminish the therapeutic effect of Gastrointestinal agent

Monitor for decreased efficacy and worsening of EPS, rigidity, tremor, or gait disturbance

Continue the section 157

**152**. Does the patient prescribed with Dexamethasone:

Body fluid retention, hypertension, depression, euphoria, bradycardia, cardiac arrhythmia, fungal infection, weight gain, hyperglycemia

Refractory to first-line agents or history of nausea/vomiting following low-emetic-risk regimens

Prophylaxis of delayed emesis (high-emetic-risk chemotherapy regimen) or alternative to NK-1 RA

Renal Failure: Severe impairment

Hepatic: Moderate and severe impairment

Anticholinergic agents

Antipsychotic agents

CNS Depressants (SSRIs, TCA)

QT-prolonging agents

**153.** Does the dose is in the range:

**High emetic risk Cisplatin and other**: Oral, IV: 12mg OD with Fosaprepitant NK1RA/ 5HT3 RA and with or without Olanzapine on day of chemo followed 8mg IV, Oral OD from day 2 to 4.

If with Rolapitant given: Oral, IV: 8 mg twice daily on days 2 to 4

If NK1 receptor antagonist not used: Oral, IV: 8 mg twice daily on days 2 to 4

**High emetic risk (AC** combination): In combination with Aprepitant, Fosaprepitant, NEPA, Fosnetupitant/palonosetron: Oral, IV: 12 mg

In combination with Rolapitant: Oral, IV: 20 mg

If NK1 receptor antagonist not used: Oral, IV: 20 mg

**Post-Chemo** dexamethasone not recommended alternative agent is or are recommended

If not, recommend

**Moderately carboplatin based**

**Day of chemo**: In combination with Aprepitant, fosaprepitant, NEPA, or Fosnetupitant/palonosetron: Oral, IV: 12 mg

In combination with Rolapitant: Oral, IV: 20 mg

**Post Chemo-** Prophylaxis is not required

**Non-carboplatin-based regimens**

**Day of chemo**: Administer prior to chemotherapy and in combination with a 5-HT3 receptor antagonist: Oral, IV: 8 mg

**Post Chemo**: Oral, IV: 8 mg on days 2 and 3

**Low emetogenic risk**: Oral, IV: 4 to 8 mg administered as a single agent in a single dose prior to chemotherapy; prophylaxis is not necessary on subsequent days

**154**. Does the dose is in the range:

If not

Concern the prescriber and recommend

**155.** Does the Dexamethasone concomitantly prescribed with:

- Nifedipine
- Hormonal contraceptive
- Doxorubicin
- Fluoroquinolones
- Amiodarone
- Tramadol/ Fentanyl
- NSAIDS
- Aprepitant/ Fosaprepitant (NK1)
- Fluconazole
- Insulin Lispro/Recombinant

May result in decreased Nifedipine exposure

May result in decreased plasma levels of hormonal contraceptive and prolonged dexamethasone effect

May result in reduced Doxorubicin exposure.

Fluoroquinolones increased risk of tendonitis tendon rupture; risk is more above 60 ages.

May result in decreased amiodarone exposure and reduced efficacy

May result in reduced Tramadol/ Fentanyl exposure

Oral dexamethasone and NSAID use together may result in increased risk of gastrointestinal ulcer

May result in increased dexamethasone exposure

May result in increased exposure to the glucocorticoid and increase the risk for toxicity

Insulin lispro, recombinant may result in may decrease blood glucose lowering effect of insulin lispro

Consider Alternate antihypertensive

Consider alternative method of contraceptives, use OC after 28 days of discontinuation of Dexamethasone

Discontinue quinoline if of pain, swelling observe

Avoid concomitant use

Monitor the serum concentration of Amiodarone

Monitor the efficacy of drug and sign of opioid withdrawal syndrome, respiratory depression or adjust dose based on dexamethasone use

Monitor the sign of bleeding

Careful monitoring required, enzyme inhibition persist for 4 to 5 days after discontinuation of Fluconazole

Reduced to 12 mg on day 1 and 8 mg on days 2 to 4

Adjust dose and/ or monitor the blood glucose level frequently

**155.** Does the Dexamethasone concomitantly prescribed with:

- Phenobarbital
- Clozapine

May result in decreased dexamethasone effectiveness

May result in reduce clozapine exposure and decrease efficacy

Monitor the efficacy of Clozapine and adjust dose of clozapine based on the dexamethasone use

Monitor the dexamethasone effectiveness, dose increase may require

**156.** Does the patient has:

Systemic fungal infection or hypersensitive to Dexamethasone

If yes

Concern prescriber, and avoid use

**157.** Does the patient is prescribed with Lorazepam

Continue the section 162

Tiredness or fatigue, dizziness, weakness, drowsiness, confusion, depression, memory loss or impairment, trouble maintaining balance

**BZD: Lorazepam**

If yes, Monitor possible ADRs

If No

**Prevent anticipatory:** 0.5 to 1 mg oral tablet by mouth once the night before chemotherapy, or the next day approximately one to two hours before chemotherapy.

**After chemotherapy:** 0.5 to 1 mg oral tablet by mouth every 6-8 hours as up to 2mg.

**158**. Does the dose is the range:

If not, Advise in recommended range

**159.** Does the patient is concomitantly using these drugs with ondansetron:

May enhance the adverse effect of other CNS depressant

May enhance CNS depressant effect of CNS depressant

CNS depressants

Magnesium sulfate

Metronidazole

Levocetirizine

Olanzapine

Opioid antagonist

Pregabalin

Monitor therapy, or dose reduction may require

May increase adverse effect of Olanzapine: hypotension, respiratory or central nervous system depression, and bradycardia

May increase the CNS depressant effect of Opioid antagonist

If necessary to use together limit the duration and dosage of the formulation.

Pregabalin and CNS depressants may result in respiratory depression

Monitor ADRs, start Gabapentin at low dose, discontinue the drug if necessary.

Avoid use

Hypersensitivity to benzodiazepines, acute narrow-angle glaucoma, propylene glycol, or benzyl alcohol, hypersensitivity to polyethylene glycol, sleep apnea severe respiratory insufficiency myasthenia gravis.

**160.** Does the use of lorazepam is contraindicated

**161.** Does the patient has:

Use with caution, or low dose may require

Injection is not recommended

**Hepatic impairment**: Severe impairment and/or encephalopathy

**Both hepatic and renal failure** condition

**Gastro-protectant drugs Acid control:**

Continue the section 165

**162**. Does the patient prescribed with PPI:

If yes, Continue and observe for the

Abdominal pain, diarrhea, flatulence, headache

If No

- Pantoprazole: IV or orally 20-40mg, 1-2 doses
- Rabeprazole: 20 mg orally 1-2 doses
- Esomeprazole: 20 to 40 mg orally 1-2 doses
- Lansoprazole: 15 to 30 mg orally once daily
- Omeprazole: 20 to 40 mg orally 1-2 doses

**163.** Does the dose in this range:

If not

Concern prescriber and recommend

**164.** Does the PPI concomitantly prescribed with:

- Methotrexate
- Capecitabine
- Ketoconazole
- Levothyroxine
- Iron
- Ampicillin
- Fluconazole

Increased concentration of methotrexate and methotrexate toxicity

PPI reduce the GI absorption and decrease the bioavailability of these drugs

Increase the plasma concentration of PPI

Monitor the Efficacy of antimicrobials

Discontinue the PPI or adjust oral Capecitabine to parenteral fluorouracil regimens

Monitor for myalgia and bone pain or to avoid toxicity discontinue PPI in Methotrexate therapy

Consider monitoring the patient for iron efficacy if Pantoprazole is being used concurrently

Carefully monitor the patient, enzyme inhibition effects of Fluconazole seen up-to 4 to 5 days of discontinuation

Administer Ketoconazole with an acidic beverage and monitor antifungal activity closely. An increase in ketoconazole dose may be indicated

Administer Levothyroxine 4 hours before or after PPI, monitor patients appropriately

**165.** Doses the patient prescribed with H_2_RB:

If yes, Continue and observe for the ADRs

If No

Abdominal pain, constipation, diarrhea, headache

Go to the section 168

**166**. Does the dose in this range:

- Ranitidine: IV 50mg Orally 150mg, 1-2 doses
- Famotidine: 10-20mg Orally, 1-2 doses
- Cimetidine: 200-400mg orally, 1-2 doses

Concern prescriber and advice

If not

**167**. Does the H2RB concomitantly prescribed with:

- Alprazolam
- Neratinib
- Ketoconazole
- Itraconazole
- Tramadol
- Fentanyl
- Clozapine
- Amiodarone
- Domperidone
- Chloroquine
- Clarithromycin
- Levofloxacin
- Metronidazole
- Apomorphine
- Fluoxetine
- Glipizide/Glimepiride
- Warfarin
- Dicumarol
- Aspirin
- Diltiazem
- Theophylline

Increased alprazolam exposure

H2RB reduce absorption and exposure of these drugs

Increased tramadol exposure and increased risk of respiratory depression

Increased risk of hypoglycemia.

Increased risk of bleeding

Increased risk of QT-interval prolongation

Reduced salicylate plasma levels and decreased antiplatelet effect of aspirin

Increased diltiazem concentrations

Increase theophylline exposure and toxicity

Advice dose reduction of Alprazolam

Use with caution

Monitor torsade’s de pointes, and ventricular arrhythmias, ECG

Monitor for respiratory depression, serotonin syndrome adjust dose based on H2RB use

Administer Tyrosine Kinase Inhibitor TKI at least 2 hours before or 10 hours after the H2RB administer

Antifungal agents given with an acidic beverage, monitor antifungal activity of them.

Monitor the patient for worsening of glycemic control

Monitor risk of bleeding, PT, INR level, prefer famotidine

Monitor blood pressure, heart rate, prefer Famotidine

Monitor Theophylline serum concentrations, nausea, vomiting, palpitations, seizures. Adjust dose based on Ranitidine use

**168.** Does the patient using Antacid:

If Yes, Observe for Any side effects

If No

Loss of appetite, diarrhea, change in taste, nausea, vomiting, headache, constipation, fecal discoloration, dizziness

Go to the section 172

**169.** Does the dose is in this range:

- Magnesium hydroxide: Oral 400 mg/5 mL, **5** to 15 mL, 1 to 4 doses
- Aluminium Hydroxide: Oral 640 mg 5 to 6 doses after meals and at bedtime
- Calcium carbonatenate: Oral 1000mg 1 to 4 doses
- Sodium bicarbonate: Oral 325 mg to 2 g 1 to 4 doses

**Combinations**

- **Oral:** Tablet: Aluminium hydroxide 200mg+ magnesium hydroxide 200mg+ simethicone 25mg 1 to 4 doses, **Suspension**: Aluminum hydroxide 200- 400mg+ magnesium hydroxide 200- 400mg+ simethicone 20-40 mg per 5 mL, 10 to 20 mL between meals, at bedtime
- Oral: Suspension: Aluminum hydroxide 291mg+ magnesium hydroxide 98mg+ Oxetacaine 10mg per 5ml, 1 to for doses before meal

If not

Concern prescriber and correct dose/frequency

**170.** Does the antacid concomitantly prescribed with:

- Antipsychotic Agents
- Captopril
- Chloroquine
- Corticosteroids
- Hyoscyamine
- Iron Preparations
- Oral antifungal
- Levothyroxine
- Tetracyclines
- Ursodiol
- Ascorbic Acid
- Multivitamins/Iron, Folate (with ADE, K or Fluoride)

Antacid reduces the absorption of these drug

Increase the absorption of Aluminum Hydroxide

Aluminum contenting antacid used with interacting drug separate at least 2 hours apart. monitor for toxic effects of Aluminium

Separate the administration of antacids and antacid interacting drug at least 2 to 4 hours, and monitor the therapy

**171**. Check if the patient has:

Renal impairment

Aluminium containing antacid may worsen the renal function

Avoid use

- Sucralfate
- If no

Go to the section 176

**172**. Dose the prescribed with ulcer protectant:

Ask patients about ADRs: Constipation, dyspepsia, dysphagia, nausea, vomiting, diarrhea, abdominal pain discomfort and palpitations

**173**. Does the dose is in this range:

Concern prescriber and adjust dose and frequency

If not

- Sucralfate: Oral tablet 1gm or Suspension 1gm/10ml, 1 to 4 doses

**174**. Does the Sucralfate concomitantly prescribed with:

Sucralfate reduces the oral absorption of the concomitantly used drugs

- Fluroquinolones
- Oral Antifungal
- Warfarin
- Levothyroxine
- Furosemide

Advise patient keep gap of at least of 2 hours between Sucralfate and interacting drug, monitor the therapeutic effectiveness of the individual drugs

- Diabetes
- Chronic kidney disease

Use with precaution proper monitoring is required

**175**. Check if the patient has:

**Others: Laxative**

Monitor for ADRs: Diarrhea, electrolytes imbalance, intestinal malabsorption, abdominal cramp, pneumonitis due to inhalation of mineral oil, hypotension, abnormal heart rate

- Lactulose
- Liquid Paraffin
- Sodium Picosulfate+ Liquid Paraffin + Milk of Magnesia

Go to the section 180

**176**. Does the patient prescribed with Laxative

If yes

If No

- Oral: Lactulose solution 10 to 20 g (15 to 30 mL, Max 40g (60) in a day ((effect may not see before 24 to 48 hours))
- Oral: Liquid Paraffin 15 to 45ml daily at bed time
- Sodium Picosulfate (3.33mg) + Liquid Paraffin (1.25ml) + Milk of Magnesia 3.75(3.75ml)

**177**. Does the dose is in this range:

If No

If not recommend in the range

**178**. Does the Laxative concomitantly with

- Iron supplement/ preparation containing iron
- Dexamethasone
- Aluminium hydroxide

Milk of magnesia may reduce the oral bio-availability/ absorption these agents

Separate the dose of these drug with Milk of magnesia at least 2 hours

Monitor the therapeutic effect of the Lactulose

Avoid use of liquid paraffin and Magnesium hydroxide

May decrease the therapeutic effect of Lactulose

- Severe renal failure
- Diabetes

**179**. Check if the patient have:

Avoid use of lactulose in patients requiring the low galactose diet

**Treatment of Neutropenia**

**180**. Does the patient prescribed with G-CSF:

### Bone pain, headaches, leukocytosis, thrombocytopenia, bruising and bleeding, hypersensitivity reaction, fever, cough, dyspnea

- Filgrastim
- Pegfilgrastim

If No

If Yes, then observe for the ADRs

Go to the section 184

- Filgrastim SC 5mcg/Kg
- Pegfilgrastim SC 6mg

**181**. Does the dose is in this range

Severe peripheral neuropathy may result due to concomitant use of Filgrastim

Don’t administer filgrastim within the time period of before and after 24 hours of chemotherapy

If not followed, suggest the nurse and physician

Don’t administer filgrastim within the time period of before and after 24 hours of chemotherapy whereas 14 days before for Pegfilgrastim and monitor the patients for possible ADRs.

**182**. Does the G-CSF concomitantly prescribed with:

- Cyclophosphamide
- Bleomycin
- Topotecan
- Vincristine

Chemotherapy pulmonary toxicity risk may increase by the G-CSF

**183**. Monitor for:

CBC with differential and platelets prior to chemotherapy and twice weekly during growth factor treatment.

If not, recommend

**Treatment for Anemia:**

Monitor for ADEs: Diarrhea, electrolytes imbalance, intestinal malabsorption, abdominal cramp, pneumonitis due to inhalation of mineral oil, hypotension, abnormal heart rate, hypophospthemia risk in iv iron, skin discoloration, ESAs may increase risk of the tumor growing, heart failure, thromboembolism, hypersensitive reaction, lungs injury,

- Erythropoiesis-stimulating agents (ESAs)
- Iron supplements
- Vitamin B12 and B9
- RBCs transfusion

**184**. Does the patient is prescribed with supplement:

If yes

If No

Go to the section 187

**185**. Does the dose is in this range:

- Erythropoiesis-stimulating agents (ESAs): Hb<10gm/dl with symptoms or <8gm without symptoms recommend ESAs, Epoetin alfa 40,000 IU once weekly, and Darbepoetin alfa 500 μg once every 3 weeks,
- Iron supplements: Ferrous sulphate 200mg 2-times/ day, elemental iron not more than 100mg/day, IV Iron Sucrose injection 100-200mg, 2-3 times/ week, Vitamin A and zinc addition with iron have effective treatment
- Vitamin B12 and B9: B12; oral 25 to 100 mcg, maximum 1000 to 2000mcg day, Deep IM; 1000mcg once/ week, or 3times/ week or once/ month.

B9: Capsule; 5 to 20mg/day, Tablet: 400 to100mcg, Injection; 5mg/ml(10ml)

- RBCs transfusion; Severe condition, if Hb<8gm/dl, consider infuse and maintain Hb>7gm/dl, hydrocortisone will be administered to prevent reaction.
- Other Vitamin A 600mcg, and vitamin C 2000mg

If No

Recommend

Iron preparation may diminish the serum concentration of these drugs Levothyroxine

**186**. Does the supplement concomitantly prescribed with:

- Antacids
- Levothyroxine
- Quinolones
- Tetracyclines
- Omeprazole
- Doxycycline
- Erythromycin
- Bleomycin
- Antacids

May diminish the oral absorption of Iron

Iron decreases the oral absorption of Tetracycline and vice-versa

May diminish the oral absorption of Cyanocobalamin

Monitor the therapy efficacy or separate the dose intake

Oral dose of these drugs separates at least for 4 hours

Advice alternative acid lowering agents H2RB, or separate the dose for 2 hour or IM route of B12 suggested

Avoid use together or monitor for seizure, coma, encephalopathy

High dose of Ascorbic acid may increase the Aluminium toxicity

Ascorbic acid may decrease the efficacy of these drugs

**Treatment of Fever and Pain**

**187**. Does the patient prescribed with Anlgesic and Antipyretic

### Anemia, thrombocytopenia, tachycardia, palpitation, periorbital edema, allergic reactions, hepatic enzyme increase, respiratory disorder,

- Paracetamol
- Paracetamol+ Tramadol
- Naproxen
- Pregabalin

If No

If Yes, then observe for the ADRs

Go to the section 192

Oral dose of these drugs separates at least for 2 hours before or 4 hours after the Tetracycline

If suspected interactions discontinue Ascorbic acid

- **Paracetamol**: Initial: 325 to 500 mg every 4 hours or 500 to 1,000 mg every 6 hours
- **Paracetamol+ Tramadol**: 325+37.5mg PO OD to TID
- **Naproxen**: Initial: 200 to 400 mg OD, followed by 200 mg every 8 to 12 hours as needed; maximum dose: 400 mg in any 8- to 12-hour period or 600 mg in a 24-hour period.
- **Diclofenac:** 100 to 150 mg/day in divided doses (immediate release and delayed release [given in 2 to 4 divided doses], or extended-release OD dose, may administer 100 mg as an initial loading dose followed by a maintenance dose; maximum dose (after day 1): 150 mg/day.
- Oral dosage form should be taken after meal with plenty of water
- **Pregabalin:** For neuropathic pain/ Peripheral neuropathy 50-100mg not more than 300mg/ day in combination with vitamin B complex

**188**. Does the dose is in this range

NSAIDs may enhance the antiplatelet effect of other antiplatelet agents

If not followed, suggest the nurse and physician

**189**. Check if the patient is concomitantly using

- CYP2C9 inducer/inhibitors
- Antiplatelet agents
- Digoxin
- Diuretics
- Methotrexate
- Multivitamins/Fluoride (with ADE) or ADEK, Iron or no mineral
- Tricyclic Antidepressants
- Carbamazepine
- Phenylephrine

May alter the he serum concentration of Diclofenac

**190**. Does the patient has:

Hypersensitivity, renal insufficiency, severe hepatic impairment or severe active liver disease, bleeding diatheses, peptic ulcer disease, gastritis.

If not, recommend

NSAIDs agent may increase the serum concentration of Digoxin

NSAIDs agents may reduce the efficacy of the diuretics

Monitor the therapy

Reduce the dose of NSAIDs or antiplatelet agents

Monitor the therapy

Monitor the therapy

May diminish the effect of acetaminophen; and also increase the risk of liver damage.

May enhance the antiplatelet effect of agents with Antiplatelet Properties

May diminish the effect of acetaminophen; and also increase the risk of liver damage.

Acetaminophen may increase the serum concentration of Phenylephrine

**191**. Monitor for:

CBC, edema, LFTs, RFT, BP, bleeding, bruising; GI effects

Observe the patient

**Antiallergic: Pheniramine, Chlorpheniramine, Hydrocortisone, Prednisolone**

**192.** Dose the patient prescribed with Antiallergic:

- Pheniramine
- Chlorpheniramine

Monitor the Adverse effect: hypotension, giddiness or drowsiness, blurred vision, palpitation, dryness of mouth, confusion, nervousness, somnolence, urinary retention

If yes

If No

Continue the section 196

If No

**Pheniramine:** **IV:** 22.75 to 20 mg in 1 to 2 ampules, maximum 40 mg per day, infuse slowly with NS

**Oral:** 17 to 20 mg PO, 6 times per day maximum for 7 days

**Chlorpheniramine:** Tablet 4 mg PO every 4 to 6 hours; maximum daily dose: 24 mg/day. Whereas extended-release tablet 12 mg PO, every 12 hours maximum 24 mg/ day. IV: 5 to 40 mg IM, IV, SC as a single dose; Maximum dose: 40 mg/day

**193**. Does the dose is the range:

If not, advise in recommended range

**194.** Does the Chlorpheniramine/ Pheniramine concomitantly prescribed with:

Monitor the therapy carefully

May result in increased risk of gastrointestinal irritation

Serotonergic agents may result in increased risk of serotonin syndrome.

Potassium citrate/chloride

Ipratropium (inhalation)

5HT3 Antagonist/

Mirtazapine

Amitriptyline

Opioid analgesic

CNS Depressants

CYP2D6 Inhibitors strong

Prokinetic GI agents

Magnesium sulfate

Avoid use together

May result in increased risk of paralytic ileus; increased risk of serotonin syndrome.

Monitor sign and symptoms of serotonin syndrome

May enhance the adverse effect of CNS depressant

May increase the concentration of Chlorpromazine

Anti-cholinergic drugs can reduce the effect of GI Prokinetics

May precipitate the anticholinergic action of Ipratropium

May enhance the CNS depressant effect of CNS depressant

**195.** Does the patient has:

Avoid use

Hypersensitivity to pheniramine/alkylamine antihistamines

If Yes

Monitor headache, dizziness, delayed wound healing, sleeplessness, mood change, sore throat, hypokalaemia, osteoporosis, fluid retention, impaired wound healing, hypertension, leucocytosis, euphoria, suprainfection

Continue the section 200

If No

**196.** Does the patient prescribed with Hydrocortisone/ Prednisolone:

**197.** Does the dose is in the range:

**Hydrocortisone:** IV 100 to 500 mg, in divided dose. Oral: 5 to 20 mg PO upto (4 to 6 times) per day.

**Prednisolone:** 5 to 60 mg once daily until symptoms are controlled, not more than 80 to 100mg/ day and dose tapering required

If not recommend same range 200

**198.** Does the Hydrocortisone/Prednisolone concomitantly prescribed with:

May alter the concentration of corticosteroids

May diminish the therapeutic effects of these drugs

May decrease the oral bioavailability of oral corticosteroids

Consider the dose gaps of 2 or more hours or consider the therapy modification

Antacids

Antidiabetic Agents

COVID-19 vaccine

CYP3A4 Inducers/inhibitors

Loop Diuretics

NSAIDs

Quinolones

Vitamin K antagonists

Estrogen derivatives

May increase the tendon rupture and tendonlitis effects of quinolines

May enhances the GI toxicity of NSAIDs

May enhance the hypokalemic effects of loop diuretics

May enhance the anticoagulant effects of vitamin K antagonist

May alter the serum concentration of corticosteroids

**199.** Does the patient has:

If systemic fungal infection or hypersensitive to these products

Concern prescriber, and avoid use

**200.** Does the patient is adhere to her medication

If No

Discuss with patient/ patients representee, and physician to improve her adherence to the drug

Monitor the therapy

**Abbreviation used:**

5HT3A: 5-hydroxytryptamine 3 receptor antagonist

ADRs: Adverse drug reactions

ALP: Alkaline phosphatase

AST: Aspartate aminotransferase

AUC: Area under curve

BD: Twice a day

BEP: Bleomycin, Etoposide and Platinum

BMI: Body mass index

BP: Blood pressure

BRACA: Breast cancer gene

BSA: Body surface area

BUN: Blood urea nitrogen

CBC: Complete blood count

CKD: Chronic kidney disease

CNS: Central nervous system

COVID-19: Coronavirus disease 2019

CT-scan: Computed tomography scan

CYP26D: Cytochrome P450 2D6

CYP3A4: Cytochrome P450 3A4

D5W: Dextrose 5% in water

DM: Diabetes mellitus

**Abbreviation used:**

DNS: Dextrose and Sodium chloride

DVT: Deep vein thrombosis

ECG: Electrocardiogram

EP: Etoposide and Platinum

EPS: Extrapyramidal side effects

ER visit: Emergency

G-CSF: Granulocyte - colony stimulating factor

GFR: Glomerular filtration rate

GI: Gastrointestinal

H2RB: Histamine H2-receptor antagonists

Hb: Hemoglobin

Hr: Hour

HRD: Homologous recombination deﬁciency

HTN: Hypertension

ICU: Intensive care unit

IM: Intramuscular

INR:  International normalized ratio

IV: Intravenous

Kg: Kilogram

LFT: Liver function tests

Mg: Milligram

MI: Myocardial infarction

**Abbreviation used:**

mL: Milliliter

NaCl: Sodium chloride

NEPA: Netupitant/palonosetron

NK1RA: Neurokinin-1 (NK-1) receptor antagonists

NS: Normal saline

NSAIDs: Non-steroidal anti-inflammatory drugs

OC: Oral contraceptive

OD: Once a day

PO: Per oral

PPI: Proton pump inhibitors

PT: Prothrombin time

QID: Four times a day

RFT: Renal function test

SC: Subcutaneous

SJS: Stevens-Johnson syndrome

SKM: Skeletal muscle

TID: Three times a day

ULN: Upper limit of normal

URTI: Upper respiratory tract infection

USG: Ultrasonography

UTI: Urinary tract infection

**Abbreviation used:**

VAC: Vincristine, Dactinomycin (actinomycin-D), and Cyclophosphamide

VIP: Etoposide (VePesid), Ifosfamide, and Cisplatin

Chemo: Chemotherapy

**References:**

**Primary sources**

- Sehirali S, Inal MM, Ozsezgin S, Sanci M, Atli O, Nayki C, et al. A randomized prospective study of comparison of reservoir ports versus conventional vascular access in advanced-stage ovarian carcinoma cases treated with chemotherapy. Int J Gynecol Cancer. 2005;15(2): 228-32.
- Sharma A, Kumari KM, Manohar HD, Bairy KL, Thomas J. Pattern of adverse drug reactions due to cancer chemotherapy in a tertiary care hospital in South India. Perspect Clin Res. 2015; 6(2): 109-15.
- Khandakar B, Kumar L, Kumar S, Gupta SD, Kalaivani M, Iyer V, et al. Tumour morphology after neoadjuvant chemotherapy as a predictor of survival in serous ovarian cancer: an experience from a tertiary care centre in India. Malays J Pathol. 2015; 37(2): 115-21.
- Manichavasagam M, Martin JPM, Lavanya R, Karthik S, Seenivasan P, Rajanandh MG. Prescribing Pattern of Anticancer Drugs in a Medical Oncology Department of a Tertiary Care Teaching Hospital. Ann Med Health Sci Res. 2017; 7: 1-3
- Degu A, Njogu P, Weru I, Karimi P. Assessment of drug therapy problems among patients with cervical cancer at Kenyatta National Hospital, Kenya. Gynaecol oncol res pract. 2017; 4:15.
- Langdon SP, Gourley C, Gabra H, Stanley B. Endocrine therapy in epithelial ovarian cancer. Expert Rev Anticancer Ther. 2017;17(2):109-17.
- Cortez AJ, Tudrej P, Kujawa KA, Lisowska KM. Advances in ovarian cancer therapy. Cancer Chemother Pharmacol. 2018. 81(1):17-38.
- Guduru H, Jeevangi SR, Nigudgi S, Bhandare SV. A prospective study on the prescription pattern of anti-cancer drugs and adverse drug reaction in a tertiary care hospital. Int J Basic Clin Pharmacol 2019; 8: 200-5.
- Spears N, Lopes F, Stefansdottir A, Rossi V, De Felici M, Anderson RA, Klinger FG. Ovarian damage from chemotherapy and current approaches to its protection. Hum Reprod Update. 2019; 25(6): 673-93.
- Lai CH, Vallikad E, Lin H, Yang LY, Jung SM, Liu HE et al. Maintenance of pegylated liposomal doxorubicin/carboplatin in patients with advanced ovarian cancer: Randomized study of an asian gynecologic oncology group. Journal of Gynecologic Oncology. 2020; 31(1): e5. doi: 10.3802/jgo.2020.31.e5
- Ingale NS, Motghare VM, Gawade SJ, Sontakke SD, Turankar AV. Drug utilization study and adverse drug reactions profile of drugs in patients of ovarian cancer in tertiary care teaching hospital. Int. J. Pharm. Sci. Rev. Res. 2021; 66(2): 74-79.
- Agarwal A, Baghmar S, Dodagoudar C, Qureshi S, Khurana A, Vaibhav V, et al. PARP Inhibitor in Platinum-Resistant Ovarian Cancer: Single-Center Real-World Experience. JCO Glob Oncol. 2021; 7: 506-

**Secondary sources**

- World Health Organization: Vigiaccess: https://www.vigiaccess.org/#/
- Garcia J, Hurwitz HI, Sandler AB, Miles D, Coleman RL, Deurloo R, Chinot OL. Bevacizumab (Avastin®) in cancer treatment: A review of 15 years of clinical experience and future outlook. Cancer Treat Rev. 2020; 86:102017. doi: 10.1016/j.ctrv.2020.102017.
- UpToDate: https://www.uptodate.com/contents/search
- Micromedex: https://www.micromedexsolutions.com/micromedex2/librarian/CS/056314/PFActionId/pf.HomePage

**Tertiary sources**

- NCCN Guidelines on ovarian cancer 2020: Available from: http://www.amoydxmed.com/uploadfile/2021/0421/20210421041203821.pdf.
- Ray-Coquard I, Morice P, Lorusso D, Prat J, Oaknin A, Pautier P, et al. Non-epithelial ovarian cancer: ESMO Clinical Practice Guidelines for diagnosis, treatment and follow-up††FootnotesApproved by the ESMO Guidelines Committee: August 2008, last update December 2017. This publication supersedes the previously published version—Ann Oncol 2012; 23(Suppl 7): vii20–vii26., Annals of Oncology. 2018; 29 (Sup 4): iv1-iv18.
- Armstrong DK, Alvarez RD, Bakkum-Gamez JN, Barroilhet L, Behbakht K, Berchuck A, et al. Ovarian Cancer, Version 2.2020, NCCN Clinical Practice Guidelines in Oncology. J Natl Compr Canc Netw. 2021 Feb 2;19(2):191-226.
- NCCN Guidelines on ovarian cancer 2021: Available from: https://www.nccn.org/patients/guidelines/content/PDF/ovarian-patient.pdf.
